# The *abaI*/*abaR* Quorum Sensing System Effects on Pathogenicity in *Acinetobacter baumannii*

**DOI:** 10.3389/fmicb.2021.679241

**Published:** 2021-07-12

**Authors:** Xiaoyu Sun, Zhaohui Ni, Jie Tang, Yue Ding, Xinlei Wang, Fan Li

**Affiliations:** ^1^Department of Pathogenobiology, The Key Laboratory of Zoonosis, Chinese Ministry of Education, College of Basic Medicine, Jilin University, Changchun, China; ^2^Department of Clinical Laboratory, The Second Hospital of Jilin University, Changchun, China; ^3^The Key Laboratory for Bionics Engineering, Ministry of Education, Jilin University, Changchun, China; ^4^Engineering Research Center for Medical Biomaterials of Jilin Province, Jilin University, Changchun, China; ^5^Key Laboratory for Biomedical Materials of Jilin Province, Jilin University, Changchun, China; ^6^State Key Laboratory of Pathogenesis, Prevention and Treatment of High Incidence Diseases in Central Asia, Xinjiang, China

**Keywords:** *A. baumannii*, quorum sensing, *abaI*/*abaR*, virulence, transcriptome

## Abstract

*Acinetobacter baumannii* is a Gram-negative pathogen that has emerged as one of the most troublesome pathogens for healthcare institutions globally. Bacterial quorum sensing (QS) is a process of cell-to-cell communication that relies on the production, secretion, and detection of autoinducer (AI) signals to share information about cell density and regulate gene expression accordingly. The molecular and genetic bases of *A. baumannii* virulence remains poorly understood. Therefore, the contribution of the *abaI*/*abaR* QS system to growth characteristics, morphology, biofilm formation, resistance, motility, and virulence of *A. baumannii* was studied in detail. RNA sequencing (RNA-seq) analysis indicated that genes involved in various aspects of energy production and conversion; valine, leucine, and isoleucine degradation; and lipid transport and metabolism are associated with bacterial pathogenicity. Our work provides a new insight into the *abaI*/*abaR* QS system effects on pathogenicity in *A. baumannii*. We propose that targeting the acyl homoserine lactone (AHL) synthase enzyme *abaI* could provide an effective strategy for attenuating virulence. On the contrary, interdicting the AI synthase receptor *abaR* elicits unpredictable consequences, which may lead to enhanced bacterial virulence.

## Introduction

*Acinetobacter baumannii* is a Gram-negative, clinically important, opportunistic pathogen that causes a wide range of clinical infections. *A. baumannii* infections are increasingly difficult to treat because of multidrug resistance in most strains causing infection in intensive care units (ICUs) (Wong et al., 2017). Virulence factors were used by bacteria to enable successful interaction with and subsequent adhesion to and invasion of the human host (Harding et al., 2018). Previous studies have emphasized the importance of iron acquisition, transports, cell-associated pili, lipopolysaccharides, and outer membrane proteins such as ompA, omp33, and surA1 for virulence (Smani et al., 2013; Liu et al., 2016; Vila-Farres et al., 2017), but studies of quorum sensing (QS) effects on pathogenicity in *A. baumannii* are limited. Bacterial QS is a process of cell-to-cell communication that relies on the production, secretion, and detection of autoinducer (AI) signals to share information about cell density and regulate gene expression accordingly (Rutherford and Bassler, 2012). AIs are involved in the regulation of varied biological functions, including expression of virulence gene in *Vibrio cholerae* (Herzog et al., 2019), *Pseudomonas aeruginosa* PAO1 (Li et al., 2017), *Staphylococcus aureus* (Kim M.K. et al., 2017), *Escherichia coli* (Zuo et al., 2019), and other bacteria (Miller and Bassler, 2001).

*Acinetobacter baumannii* presenting a typical QS system (*abaI*/*abaR*) has been described (Bhargava et al., 2010). The *abaI* gene encodes 183 amino acids, and this protein was predicted to function in signal transduction. The *abaR* gene encodes 238 amino acids, and this protein is an AI synthase receptor (Bhargava et al., 2010). Previous studies on *A. baumannii* had focused on the role of QS systems in drug resistance, biofilm formation, and motility (Bhargava et al., 2015; Dou et al., 2017), but studies of QS effects on pathogenicity in *A. baumannii* are poor.

In this study, we used *A. baumannii* strain ATCC 17978, which has been the most frequently used model for scientific studies over the past two decades (Harding et al., 2018). A previous study isolated and characterized the AI synthase *abaI* from *A. baumannii* M2 but failed to create an *abaR* deletion mutant for unknown reasons (Niu et al., 2008). To explore the role of the *abaI*/*abaR* QS system in drug resistance, biofilm formation, and virulence of *A. baumannii*, Δ*abaI*, and Δ*abaR* mutants of strain ATCC 17978 were created. We also made double-mutant Δ*abaIR*. The transcriptomes of wild type (WT), Δ*abaI*, Δ*abaR*, and Δ*abaIR* were determined by RNA sequencing (RNA-seq).

## Materials and Methods

### Bacterial Strains, Plasmids, and Culture Conditions

Bacterial strains and plasmids used in this study are listed in Table 1. *A. baumannii* strains were grown in lysogeny broth (LB). Antibiotics were used at the different concentrations for *E. coli* (kanamycin, 10 mg/L; ampicillin 25 μg/ml; and tellurite, 6 mg/L) and for *A. baumannii* (tellurite, 30 mg/L; and tetracycline, 50 mg/L).

**TABLE 1:** Bacterial strains and plasmids used in this study.

Strain or plasmid	Relevant characteristics	Reference or source
***Acinetobacter baumannii* strains**		
ATCC 17978 (WT)		Ayush Kumar
Δ*abaI*	WT with deletion in *abaI* gene	This study
Δ*abaR*	WT with deletion in *abaR* gene	This study
Δ*abaIR*	WT with deletion in *abaI* and *abaR* genes	This study
Δ*abaI*(pME*abaI*)	Δ*abaI* harboring pME6032, containing the *abaI* gene	This study
Δ*abaR*(pME*abaR*)	Δ*abaR* harboring pME6032, containing the *abaR* gene	This study
WT(pME6032)	WT harboring pME6032, empty vector	This study
WT(pME*abaI*)	WT harboring pME6032, containing the *abaI* gene	This study
WT(pME*abaR*)	WT harboring pME6032, containing the *abaR* gene	This study
***E. coli* strains**		
DH5α	F-φ 80*lacZ* Δ M15 Δ (*lacZYA*-*argF*) U169 A1 *recA*1 *hsdR*17(*rk*-, *mk*+)*supE*44λ-*thi*-1 *gyrA*96 *relA*1 *phoA*	TransGen
S17-1 (ATCC 47055)	Genotype: *recA pro hsdR RP*4-2-*Tc*:*Mu*-*Km*:*Tn*7,*Gm*^*S*^	Biobw
***Chromobacterium violaceum***		
CV026	Detection of C4- and C6-HSLs	Mingsheng Dong
***Agrobacterium tumefaciens***		
KYC55 (pJZ372) (pJZ384) (pJZ410)	Detection of broad range of AHLs	Mingyong Zeng
R10 (pCF218)	*Ptet*-*traR*, *Tc*^*R*^, high AHL production strain	Mingyong Zeng
**Plasmids**		
pMO130-Tel^*R*^	Suicide plasmid, *xylE*^+^, *sacB*^+^, *Km*^*R*^	Addgene
pMO130-Tel^*R*^-*abaI*-(Up/Down)	pMO130-Tel^*R*^ containing a 1 kb UP fragment (*abaI*) and 1 kb DOWN fragment (*abaI*)	This study
pMO130-Tel^*R*^-*abaR*-(Up/Down)	pMO130-Tel^*R*^ containing a 1 kb UP fragment (*abaR*) and 1 kb DOWN fragment (*abaR*)	This study
pME6032	Shuttle plasmid for genetic complementation, *Tc*^*R*^	Ke Lei Biological Technology Co., Ltd.
pME*abaI*	pME6032 containing the *abaI* gene (promoter and coding region)	This study
pME*abaR*	pME6032 containing the *abaR* gene (promoter and coding region)	This study

### Strain Construction

Strains Δ*abaI*, Δ*abaR*, and Δ*abaIR* were unmarked deletion mutants created by a previous described method for acquiring markerless deletions in *A. baumannii* with minor modifications (Amin et al., 2013). The primers used in this study are listed in Table 2. Briefly, the upstream and downstream homologous arms of the target gene were amplified and fused and then ligated into a tellurite-resistant suicide vector with the T4 ligase, pMO130-Tel^*R*^ (a generous gift from Addgene). The plasmid constructs were first introduced into *E. coli* DH5α and subsequently selected on LB agar containing 30 μg/ml kanamycin. The kanamycin-resistant colonies which carry an insertion of pMO130-Tel^*R*^ and a 2-kb amplimer corresponding to the size of the ligated upstream and downstream homologous arms of the target gene were tested by the corresponding designed primers. The resulting plasmids were used to transform into *E. coli* S17-1 and subsequently conjugate into *A. baumannii* ATCC 17978 via biparental conjugation. Exconjugants were selected on LB containing 30 μg/ml tellurite and 25 μg/ml ampicillin. *A. baumannii* ATCC 17978 harboring the inserted pMO130-Tel^*R*^-Gene-(Up/Down) construct was cultured in LB containing 10% sucrose and passaged 7 days to select for stabilized deletion of gene and loss of the *sacB* gene by a second crossover and allelic replacement. If the target gene has been deleted, the PCR of genomic DNA from these bacteria would not produce any amplimer using a primer pair that anneals to the DNA that has been deleted. Mutants were complemented with the pME*abaI* and pME*abaR* plasmids, generated by cloning the *abaI* and *abaR* genes and ligation into a shuttle plasmid vector with the T4 ligase, pME6032 (Heeb et al., 2000). Overexpressed strains were transformed by the pME*abaI* and pME*abaR* plasmids. The complemented strains and overexpressed strains were confirmed by PCR and restriction analysis of plasmids extracted from *A. baumannii* cells grown in LB medium containing 50 μg/ml tetracycline. All operations were performed in the P2 Laboratory of Basic Medical College of Jilin University, following the biosafety standard operating procedures of the Basic Medical College of Jilin University.

**TABLE 2:** Primers used in this study.

Primer name	Sequence	Product
*abaR*(*Sph*I)up-F	TATGCATGCTTACGCCACTGACTAAGAG	1,190 bp
*abaR*(*Bam*HI)up-R	GCTGGATCCCGATAAGAGACCACTAACCT	
*abaR*(*Bam*HI)dw-F	TATGGATCCTTGAAGCGTAGGTCTAATCT	1,136 bp
*abaR*(*Pst*I)dw-R	TATCTGCAGAAGGCGGTAACTGTAAGAA	
*abaI*(*Pst*I)dw-F	TATCTGCAGCGCAACTACAGCCATACT	932 bp
*abaI*(*Not*I)dw-R	TATGCGGCCGCGCCTCTTACCGACTTACG	
*abaI*-F	AAAGTTACCGCTACAGGG	435 bp
*abaI*-R	CACGATGGGCACGAAA	
*abaR*-F	TCCTCGGGTCCCAATA	310 bp
*abaR*-R	TAAATCTACCGCATCAA	
*abaI*(*Eco*RI)-F	CCGGAATTCCGGGTGGAAGCACTTGTAATGAA	654 bp
*abaI*(*Xho*I)-R	CCGCTCGAGCGGCTCATCTTGCTCGGTCATA	
*abaR*(*Eco*RI)-F	CCGGAATTCCGGCTACAAAAGCCCTAGCATT	808 bp
*abaR*(*Xho*I)-R	CCGCTCGAGCGGAAGATTAGACCTACGCTTCA	
pME6032-F	CCTCATCAGTGCCAACATA	843 bp
pME6032-R	CATACTCTGCGACATCGTA	

### Growth Curve Measurement

A single colony of strain was inoculated into 2 ml of LB and cultured with shaking (200 rpm) overnight at 37°C. The bacteria were collected by centrifugation at 4,000 rpm for 5 min and suspended in sterile saline to a turbidity comparable to a 0.5 McFarland standard. Twenty microliters was pipetted into a 96-well microtiter plate containing 180 μl of LB and incubated at 37°C. The optical density (OD)_600_ of cultures was measured at hourly intervals for up to 48 h to draw the growth curve. Tests were performed on eight individual biological replicates, in triplicates.

### Transmission Electron Microscopy

Bacterial cells (OD_450_ of 1.0) for SEM observation were harvested by centrifugation and washed three times with ddH_2_O. Bacteria were prefixed with 2.5% glutaraldehyde in 0.1 M phosphate buffer (pH 7.4). Images were captured at 120 kv with a HITACHI H-7650 transmission electron microscopy (TEM).

### Antimicrobial Susceptibility

*Acinetobacter baumannii* strains were cultured in LB liquid medium at 37°C with overnight shaking. Minimum inhibitory concentrations (MICs) for kanamycin, penicillin, streptomycin, meropenem, imipenem, ceftizoxime, cefepime, cefoperazone–sulbactam, piperacillin–tazobactam, ampicillin, tetracycline, and spectinomycin were determined on 96-well plates by the broth microdilution protocols of the Clinical and Laboratory Standards Institute, and the results of MIC testing were interpreted according to the criteria of the CLSI 2013 guidelines. All experiments were carried out a minimum of three times.

### Screening of Acyl Homoserine Lactone by *Chromobacterium violaceum* CV026 and *Agrobacterium tumefaciens* KYC55

*Acinetobacter baumannii* can produce acyl homoserine lactone (AHL) signal molecules with different chain lengths (Erdonmez et al., 2017); it is essential to use biosensors that detect a broad range of AHLs. *C. violaceum* CV026 specific for short-chain AHLs (C4–C6 AHL molecules) and *A. tumefaciens* KYC55 specific for long-chain AHLs (C8–C14 AHL molecules) were utilized for screening AHLs producing bacterial strains. By the presence of short-chain AHLs, CV026 produces purple pigments. By the presence of long-chain AHLs, a green color is observed for *A. tumefaciens* KYC55 (Erdonmez et al., 2017). Screening of the *A. baumannii* for production AHLs was carried out by agar plate diffusion assay with minor modifications (Lade et al., 2014; Erdonmez et al., 2017). Briefly, QS reporter strains were cultured in LB agar plates containing the antibiotics kanamycin 20 μg/ml for *C. violaceum* CV026 and spectinomycin 50 μg/ml and tetracycline 4.5 μg/ml for *A. tumefaciens* KYC55. The plates were incubated at 28°C for 24 h. As a visualizing agent, 40 μg/ml of X-gal was incorporated into the LB medium used for *A. tumefaciens* KYC55. For determining the production of acyl homoserine signal molecules, *A. baumannii* and mutants and biosensor *C. violaceum* CV026 and *A. tumefaciens* KYC55 strain were inoculated side by side such that they had a 0.5-cm gap between them. *C. violaceum* CV026 and *A. tumefaciens* KYC55 were assessed to be positive or negative according to the color changes in biosensor strain.

### Surface Motility Assay

The motility test was performed according to the method described previously with minor modifications (Clemmer et al., 2011). Briefly, the medium used for surface motility assay was tryptone broth [10 g/L tryptone (OXOID) and 5 g/L NaCl] supplemented with 0.3% (wt/vol) Noble agar (BD). Plates were prepared and inoculated with bacteria from an overnight culture in LB agar (1.5%, wt/vol) plates at 37°C with a sterile toothpick. All assays were carried out in triplicate in a minimum of three independent experiments. After incubation at 30°C for 12–14 h, the zone of motility at the agar/Petri dish interface was observed.

### Crystal Violet Biofilm Assay

The biofilm-forming ability test was performed in accordance with the method described previously with minor modifications (Kaplan et al., 2012). Briefly, a few single colonies were suspended in sterile saline to a turbidity comparable to a 0.5 McFarland standard. The suspension was under vortex movement for 1 min; 20 μl was pipetted into a 96-well microtiter plate containing 180 μl of LB and incubated for 24 h at 37°C. For crystal violet staining, the wells were rinsed with phosphate-buffered saline (PBS) to exclude loosely adherent cells and then stained for 30 min with 200 μl of 1% crystal violet. The wells were then rinsed with water and dried at room temperature. The amount of biofilm was quantitated by destaining the wells with 200 μl of 33% acetic acid and then measuring the OD of the solution in a microplate spectrophotometer set at 595 nm. Tests were performed on 10 individual biological replicates, in triplicates. The differences between parent and mutant strains were calculated, and values returning a *P*-value of < 0.05 from Student’s *t*-test were taken as significant.

### Serum Bactericidal Assay

The serum resistance experiment was performed in accordance with the method previously described with minor modifications (Harris et al., 2013). Briefly, 100 μl mid-log-phase *A. baumannii* culture (a bacterial titer of approximately 1 × 10^5^ CFU) was mixed with 900 μl of either normal human serum (NHS) or heat-inactivated serum (heated at 56°C for 30 min). The mixtures were incubated at 37°C, and aliquots of 100 ml were removed from the culture at 1 h for the determination of bacterial counts. The number of surviving CFUs was determined by plating in triplicate. The results were expressed as percentage of survival, with 100% being the number of viable bacteria grown on brain heart infusion agar plates.

### Virulence in *Galleria mellonella*

*Galleria mellonella* has been known as a good model system to study *A. baumannii* pathogenesis (Peleg et al., 2009). The survival of ATCC 17978 and mutants in *G. mellonella* was measured as previously described (Peleg et al., 2009). An inoculum of 10^6^ CFU bacteria was injected into *G. mellonella* larvae. After injection, the larvae were incubated at 37°C in darkness, and death was assessed at 24-h intervals over 7 days. The experiment was performed on 10 individual biological replicates, in triplicate. Statistical analysis was carried out with GraphPad Prism 6 to produce Kaplan–Meier survival curves. The statistical significance of differences between parent and Δ*abaI*, Δ*abaR*, and Δ*abaIR* mutant strain survival curves was calculated with a log rank test. *P*-values of <0.05 were considered significant.

### Murine Model of Pneumonia

Eight- to 10-week-old BALB/C mice were obtained from Jilin University. Mice were kept in a sterile environment at Jilin University and maintained according to standard procedures. All research was conducted in compliance with the institutional guidelines. The principles in the ARRIVE guidelines and the Basel declaration)^1^ have been considered when planning the experiments. Models of pulmonary infection were performed as previously described (Geisinger and Isberg, 2015). Briefly, infections were initiated by intraperitoneal injection of approximately 1.2 × 10^8^ CFU of bacteria suspended in 100 μl of PBS into groups of mice (10 mice per group for survival studies; 5 per group for analyses of bacterial counts).

### Quantitative Bacteriology

To assess bacterial burden, the lung and spleen were aseptically operated and homogenized in 1 ml of sterile PBS using tissue homogenizers. Cultured on brain heart infusion agar plates were 100 μl of the homogenates to quantify the bacterial load of *A. baumannii* in the respective organs.

### RNA-Seq and Analysis

RNA was extracted from three biological replicates of each strain with a GeneMark Total RNA Purification Kit. RNA libraries were prepared and sequenced with Illumina HiSeq 2000 at the Beijing Genomics Institute (BGI). Sequences were mapped onto the ATCC 17978 genome (accession no. cp018664.1) using Bowtie 2. Differentially expressed genes (DEGs) were identified using the DESeq2 package (Bioconductor). Genes were deemed as differentially expressed if they presented a log2-fold change greater than 1 or less than −1 and if *P-*value (*P*-adj) was less than 0.05 in the mutant strain compared to the WT strain. Cluster of orthologous groups (COG) enrichment analysis was performed by dividing the percentage of genes upregulated or downregulated for each category by the percentage of genes in that category across the whole genome (Tatusov et al., 2000; Galperin et al., 2015). MultiExperiment Viewer version 4.9.0 was used to perform hierarchical clustering and heat map visualization (Howe et al., 2011). Gene Ontology (GO) and Kyoto Encyclopedia of Genes and Genomes (KEGG) pathway enrichment analyses, based on R software, were applied for the identification of pathways in which DEGs were significantly enriched. The GO and KEGG pathway analyses of the DEGs were conducted through the clusterProfiler package in R software (Yu et al., 2012). A *P*-value of < 0.05 was considered to have statistical significance and to achieve significant enrichment. Quantitative reverse transcription (qRT)-PCR was performed on the 7300 Plus Real-Time PCR System (Applied Biosystems) using a standard protocol from the FastStart Universal SYBR Green Master (Roche, Basel, Switzerland). Gene expression levels were quantified by using the 2^–Δ^ ^Δ^ ^*Ct*^ method with endogenous controls (16S). The primers used for qRT-PCR assays are described in Table 3. All qRT-PCR assays were repeated 3×. The RNA-seq data obtained in this study were submitted to GEO, with accession number GSE173396.

**TABLE 3:** Real-time quantitative PCR primers.

qRT-PCR	Sequence
AUO97_RS01130-F	CGCAACGCCATTACTA
AUO97_RS01130-R	TTGTTTATCGCATCCTG
AUO97_RS01135-F	TTGCTCCACCCACATA
AUO97_RS01135-R	TTGGCGTAACTTCACTT
AUO97_RS08710-F	ATGCCGAGTTTGCTTA
AUO97_RS08710-R	AACACGCTGTGAATCTTT
AUO97_RS13835-F	CGGTGCTTGATGTGCT
AUO97_RS13835-R	AAATGCGATAACGTGGA
AUO97_RS17125-F	GTCTACGCCGCTCTGT
AUO97_RS17125-R	AGGTTATTGAAGGTGGG
AUO97_RS17130-F	CCACATACGCCTTGCT
AUO97_RS17130-R	CCTCGGGAGATTCATT
AUO97_RS17135-F	TTTTGGGCATACTGACTTT
AUO97_RS17135-R	CTTCTGGACTCGGTAATGT
AUO97_RS17140-F	CCTTTGGTGCCGTAGA
AUO97_RS17140-R	ACCCGAACCTCACAGAC
AUO97_RS17145-F	GCGTTCCCAAGCCTCA
AUO97_RS17145-R	CTCGGTGATTACGATGGATG
AUO97_RS17160-F	CACCCAACCCACTGAA
AUO97_RS17160-R	AGCGTATGTGAGCCAAG
16S_qF	CAGCTCGTGTCGTGAGATGT
16S_qR	CGTAAGGGCCATGATGACTT

## Results

### The Mutants Showed Differences in Growth Characteristics and Morphology

**FIGURE 1:**
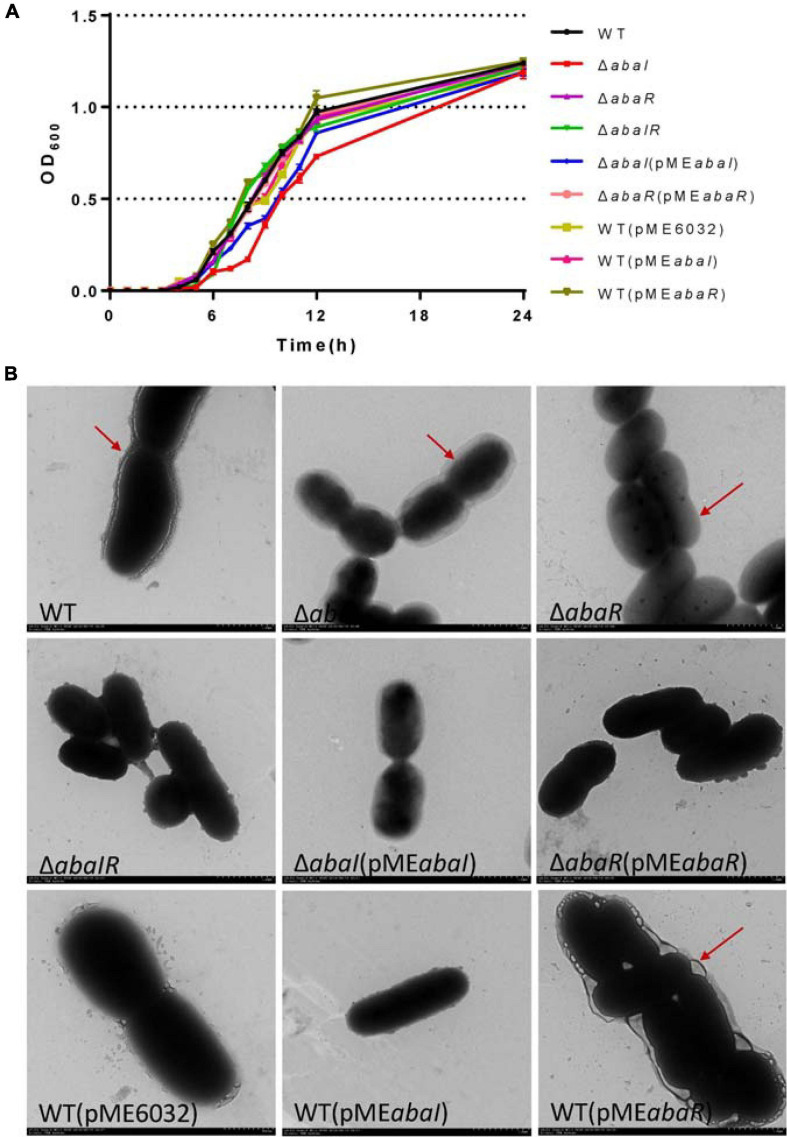
**(A)** Growth curve analysis of *A. baumannii* and mutants in broth. **(B)** TEM images of the targeted bacteria. Cells were observed with a HITACHI H-7650 TEM operated at 120 kV. Scale bar = 500 nm.

To test the role of the *abaI*/*abaR* QS system in the *A. baumannii* growth curve, we determined the OD of the culture over time. The growth of the Δ*abaR* and Δ*abaIR* mutants did not differ from that of the parent strain (Figure 1A). The complemented strain Δ*abaR* (pME*abaR*) and overexpressed strains WT(pME*abaI*) and WT(pME*abaR*) also showed growth profiles similar to those of the WT (Figure 1A), suggesting that the gene *abaR* is not essential for *A. baumannii* growth. In contrast, the Δ*abaI* mutant showed visibly slowed growth at the logarithmic phase. The growth of the Δ*abaI* mutant was partly rescued by the expression of *abaI* via the pME6032-derived plasmid pME*abaI* (Figure 1A). The empty vector pME6032, used as a control, did not affect the growth profile of *A. baumannii* ATCC 17978 (Figure 1A). These results demonstrated that *abaI* affects the growth of *A. baumannii*. Subsequently, the morphology of the bacteria was observed by TEM. As shown in Figure 1B, WT was attached with extracellular secretions. Δ*abaI* mutant cell edges were transparent. The Δ*abaR* mutant strain’s cytoplasmic density is lower. There was no obvious change in the Δ*abaIR* mutant, but around the cell adhered partial secretions. The morphology of the Δ*abaI* mutant was partly rescued by the expression of *abaI* via the plasmid pME*abaI.* The morphology of the Δ*abaR* mutant was rescued by the expression of *abaR* via the plasmid pME*abaR*. There was no significant difference between WT and the overexpressed strain WT(pME*abaI*). The overexpressed strain WT(pME*abaR*) attached a large number of extracellular secretions. The empty vector pME6032, used as a control, did not affect the morphology of *A. baumannii* ATCC 17978. These results indicated that *abaI* is closely related to cell morphology and extracellular secretions.

### Mutants Showed Increased Susceptibility to Antimicrobials

To determine whether deletion of QS genes changed in the drug resistance, the MICs of commonly used antibiotics for strains were determined. There was a decrease in the MICs of kanamycin, gentamicin, penicillin, streptomycin, meropenem, imipenem, and ampicillin for QS gene deletions compared to WT. Drug susceptibility of Δ*abaI* and Δ*abaR* mutants was partly rescued by expressions of *abaI* and *abaR* via plasmids pME*abaI* and pME*abaR*, respectively. The overexpressed strain WT(pME*abaI*) was more resistant in cefepime and cefoperazone–sulbactam. WT(pME*abaR*) was more resistant in kanamycin, streptomycin, ceftizoxime, cefepime, cefoperazone–sulbactam, and piperacillin–tazobactam. The empty vector pME6032, used as a control, partly affects the drug susceptibility of *A. baumannii* ATCC 17978 (Table 4). These results indicated that QS affects antimicrobial sensitivity. The preliminary research of the research group showed that *abaI* and *abaR* genes positively correlated with bacterial resistance rates (Tang et al., 2020).

**TABLE 4:** Minimum inhibitory concentrations of antibiotics used in this study.

	MIC (μg/ml)								
Antibiotic	WT	Δ*abaI*	Δ*abaR*	Δ*abaIR*	Δ*abaI*(pME*abaI*)	Δ*abaR*(pME*abaR*)	WT(pME6032)	WT(pME*abaI*)	WT(pME*abaR*)
Kanamycin	8	2	4	4	4	8	8	8	16
Gentamicin	4	1	4	0.25	2	4	4	4	4
Penicillin	128	64	64	32	64	64	64	64	128
Streptomycin	32	16	16	16	32	32	32	32	64
Meropenem	1	0.25	0.5	0.25	0.5	0.5	1	1	1
Imipenem	32	16	16	8	16	16	16	16	32
Ceftizoxime	8	8	8	8	8	8	8	8	16
Cefepime	2	8	8	2	8	8	8	8	8
Cefoperazone–sulbactam	2	8	2	2	8	4	2	4	4
Piperacillin–tazobactam	8	8	8	32	8	8	8	8	16
Vancomycin	>512	>512	128	>512	>512	>512	>512	>512	>512
Ampicillin	64	32	64	32	32	32	32	32	64
Tetracycline	2	8	2	2	8	4	4	4	4
Spectinomycin	64	64	64	64	64	64	64	64	64

### Screening of Strains for AHL Production

**FIGURE 2:**
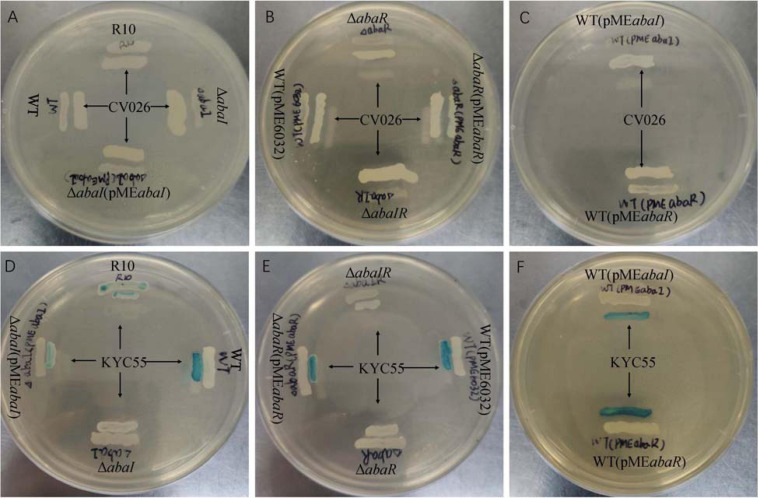
Screening of WT and mutants for AHL production using agar plate well diffusion assay with *C. violaceum* CV026 and *A. tumefaciens* KYC55 reporter strains. **(A–C)** WT, mutants and AHL positive strain R10 were tested with CV026. **(D–F)** WT, mutants and AHL positive strain R10 were tested with KYC55.

To detect the effect of the QS system on AHL of the strains, two different biosensor strains were used. As a result, no strains were found to produce AHLs based on the development of purple coloration in the CV026 reporter strain (Figures 2A–C). Δ*abaI*, Δ*abaR*, and Δ*abaIR* were not observed to produce AHLs based on the development of green coloration in the *A. tumefaciens* KYC55 reporter strain. AHL production of Δ*abaI* and Δ*abaR* mutants was rescued by expressions of *abaI* and *abaR* via plasmids pME*abaI* and pME*abaR*, respectively. The empty vector pME6032, used as a control, did not affect the AHL production of *A. baumannii* ATCC 17978 (Figures 2D–F). These results indicate that both *abaI* and *abaR* can affect AHL production and that *A. baumannii* may produce only long-chain signal molecules, not short-chain AHLs.

### Surface-Associated Motility Relies on QS

**FIGURE 3:**
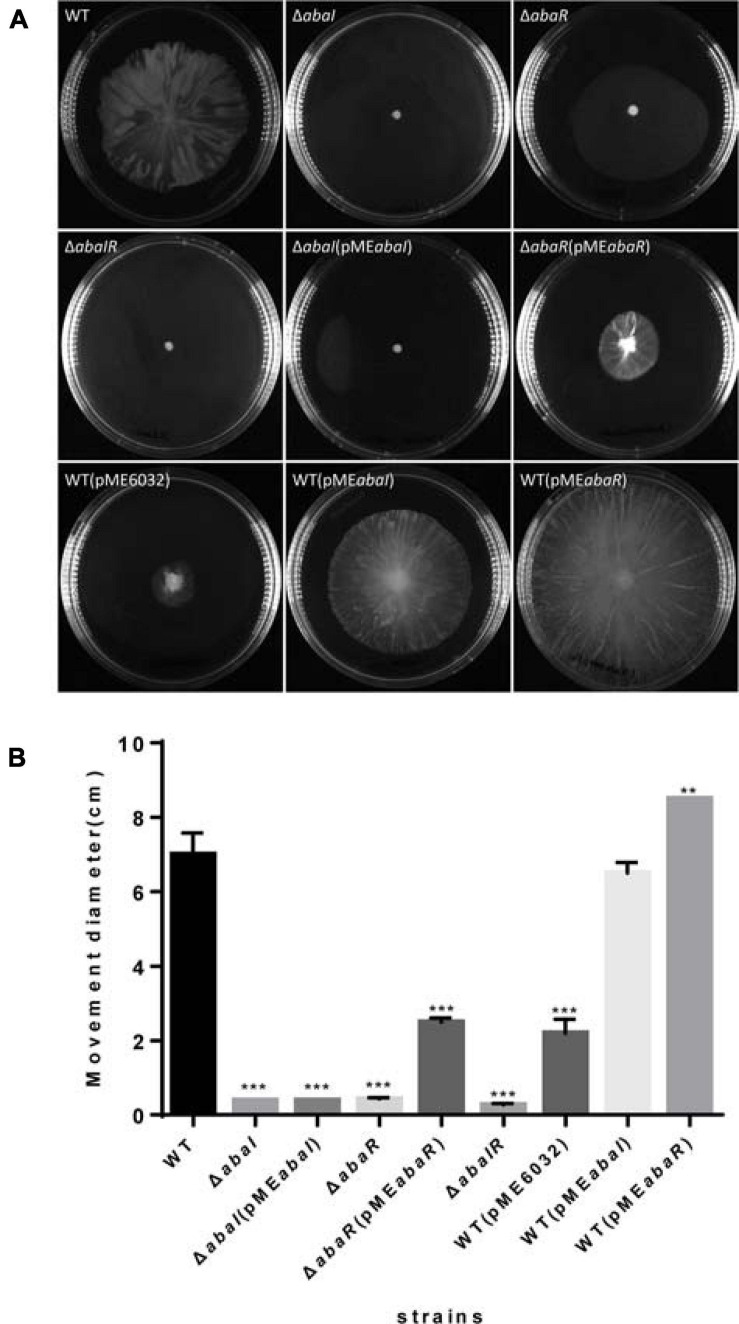
**(A)** Wild type and mutants were inoculated on the surface of a semisolid agarose plate (0.3%) and incubated for 12–14 h at 30°C. *abaI*, *abaR*, and *abaIR* mutant strains demonstrated defects in surface-associated motility compared to the parental strain. **(B)** The distance migrated (diameter) is shown for each strain, and error bars represent SD for three biological replicates. **P < 0.005; ***P < 0.001.

To explore the role of QS in motility, we tested these strains’ surface motility phenotype on LB medium supplemented with 0.3% agar. As a result, Δ*abaI*, Δ*abaR*, and Δ*abaIR* mutants compared with WT strain showed no obvious motility, motility of Δ*abaI* mutants was not rescued by the expression of *abaI* via the plasmid pME*abaI*, and motility of Δ*abaR* mutants was partly rescued by the expression of *abaR* via the plasmid pME*abaR*. The empty vector pME6032, used as a control, has an inhibitory effect on the motility of *A. baumannii* ATCC 17978. The overexpressed strain WT(pME*abaR*) compared with the WT strain showed significantly increased motility. There was no significant difference between the overexpressed strain WT(pME*abaI*) and WT in the motility (Figures 3A,B). These results indicated that the QS system affects the motility of bacteria and that the effect of *abaR* is stronger than that of the *abaI* mutant on motility, since the Δ*abaI*(pME*abaI*) could not rescue the normal phenotype in comparison with Δ*abaR*(pME*abaR*) (Figure 3A).

### Biofilm Formation

**FIGURE 4:**
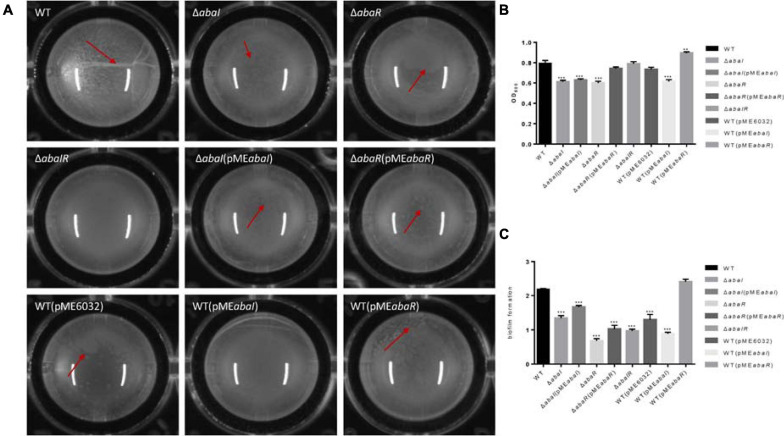
**(A)** Visual changes in biofilm formation in 96-well plates for 24 h at 37°C; images were taken after 24 h of growth. The red arrow showed the biofilm produced in the liquid–air interface, known as pellicle biofilm. **(B)** The absorbance of bacterial solution was detected by OD_595_. **(C)** Biofilm formation on plastic at 37°C as determined by crystal violet staining. Markers show the OD_595_ compared with WT in individual biological replicates. **P < 0.005; ***P < 0.001.

To investigate the role of QS in biofilm formation on an abiotic surface, we cultivated the strain mutants in 96-well plates for 24 h at 37°C. As a result, filaments were formed in the culture medium of the WT strain, mutant strains Δ*abaI* and Δ*abaR* had dot-like biofilm formation on the surface of liquid, the Δ*abaIR* mutant strain had no biofilm on the liquid surface, there were dot-like biofilms on the liquid surface of Δ*abaI*(pME*abaI*) and Δ*abaR*(pME*abaR*), and they were connected into pieces. The biofilm on the liquid surface of the overexpressed strain WT(pME*abaI*) was lamellar, while the biofilm on the liquid surface of WT(pME*abaR*) was spot-like but thick (Figure 4A). The membrane at the liquid–gas interface of the WT(pME6032) was not obvious. The absorbance of the bacterial solution was detected, and the results are shown in Figure 4B; there were significant differences between the absorbance of the parental strain and mutants Δ*abaI* and Δ*abaR*; however, the mutant Δ*abaIR* showed no difference. The absorbance of Δ*abaI* mutants was not rescued by the expression of *abaI* via the plasmid pME*abaI*, and the absorbance of Δ*abaR* mutants was rescued by the expression of *abaR* via the plasmid pME*abaR*. The absorbance of the overexpressed strain WT(pME*abaR*) was significantly higher than that of the WT strain and WT(pME6032). The biofilm-forming ability of the strains was determined by crystal violet biofilm assay, and the differences between the WT and mutant strains were calculated. Compared with that of WT strains, the biofilm formation of all strains was significantly decreased, except for that of the overexpressed strain WT(pME*abaR*), which was significantly higher than that of the WT strain (Figure 4C). WT(pME*abaI*) produced less biofilm in comparison with its control WT(pME6032) harboring the plasmid alone, which may be due to the influence of plasmid pME*abaI* on the secretion of some proteins in the WT strain. The results indicated that QS system affects the pellicle biofilm formation in the liquid–air interface.

### Serum Killing

**FIGURE 5:**
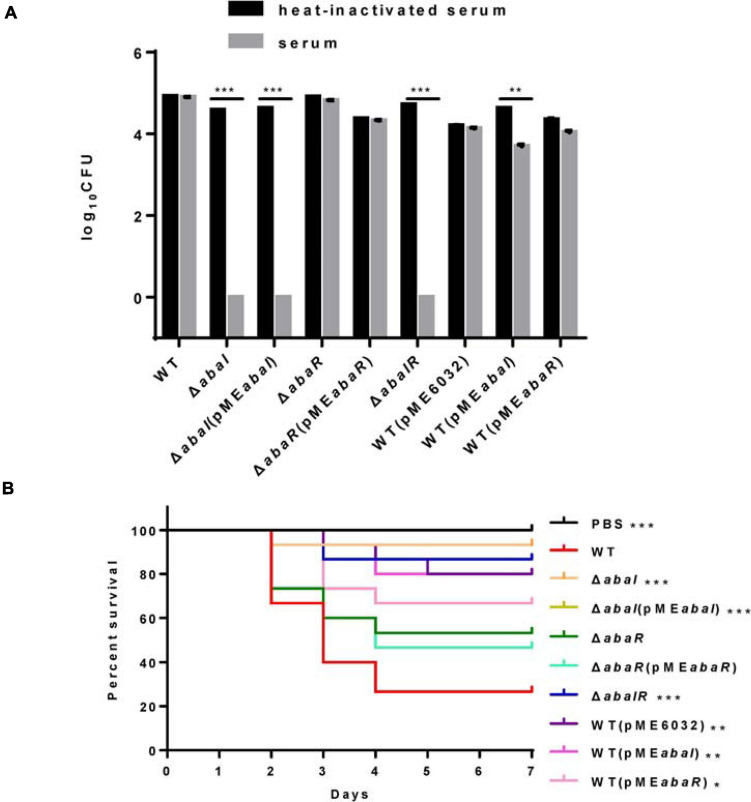
**(A)** Sensitivity of strains to NHS. Viable bacterial counts were determined after 1 h of incubation at 37°C with rotation. Data are presented as percentage of survival, with 100% being the number of viable bacteria grown in heat-inactivated serum. All values are from triplicate samples and are representative of three independent experiments. **(B)** Kaplan–Meier survival curve showing the virulence of PBS, WT, and mutants in *G. mellonella*. The data show the percentage of survival (*n* = 30) of *G. mellonella* after inoculation with 10^6^ CFU of bacteria. Survival curves were compared using the log-rank (Mantel–Cox) test. *P < 0.05; **P < 0.005; ***P < 0.001.

Serum sensitivity has been involved in the toxic mechanisms of *A. baumannii*; to elucidate the virulence of strains, we compared the serum sensitivity of strains to NHS. As shown in Figure 5A, WT, *abaR*, and WT(pME*abaR*) survived after incubation in serum, whereas Δ*abaI*, Δ*abaI*(pME*abaI*), and Δ*abaIR* were entirely killed after 1 h at 37°C. The serum sensitivity of Δ*abaI* mutants was not rescued by the expression of *abaI* via the plasmid pME*abaI*, and the serum sensitivity of Δ*abaR* mutants was rescued by the expression of *abaR* via the plasmid pME*abaR*. The empty vector pME6032, used as a control, did not affect the serum sensitivity of *A. baumannii* ATCC 17978. These results indicated that WT and Δ*abaR* were highly resistant to the serum; in contrast, Δ*abaI* and Δ*abaIR* mutants were much more serum sensitive, showing a significant difference (*P* < 0.001).

### QS Plays a Role in Virulence in *G. mellonella* Infection Models

Quorum sensing controls the production of virulence factors in many bacterial species and is regarded as an attractive target to combat bacterial pathogenicity (Mukherjee et al., 2018). To explore whether QS genes are an important virulence factor determinant for *A. baumannii* in *G. mellonella*, we assessed the virulence of the QS mutant strains compared to the isogenic parent strain ATCC 17978. Δ*abaI* and Δ*abaIR* mutants were completely avirulent in this assay, and the Δ*abaIR* mutant was slightly more pathogenic than the Δ*abaI* mutants but less pathogenic than the Δ*abaR* mutants, while the Δ*abaR* mutant remained fully virulent, killing *G. mellonella* larvae as toxic as the WT (Figure 5B). Consistent with this finding, Laura Fernandez-Garcia et al. (2018) found that injection of *G. mellonella* larvae with the *A. baumannii* ATCC 17978 strain caused higher mortality than injection with the mutant *A. baumannii* ATCC 17978 Δ*abaI*. The virulence of the Δ*abaI* mutant was not rescued by the expression of *abaI* via the plasmid pME*abaI*, and the virulence of the Δ*abaR* mutant was not rescued by the expression of *abaR* via the plasmid pME*abaR*. The empty vector pME6032, used as a control, reduced the virulence of *A. baumannii* ATCC 17978. The virulence of the overexpressed strains WT(pME*abaI*) and WT(pME*abaR*) was significantly reduced compared to that of the parent strain ATCC 17978 (Figure 5B). The virulence of strains WT(pME*abaI*) and WT(pME*abaR*) was not significantly different from that of WT(pME6032). The results indicated that the QS system plays a role in virulence in *G. mellonella* infection models and that plasmids not only affect the virulence of WT strains but also affect the virulence of complement strains.

### QS Plays a Role in Virulence in Mouse Infection Models

**FIGURE 6:**
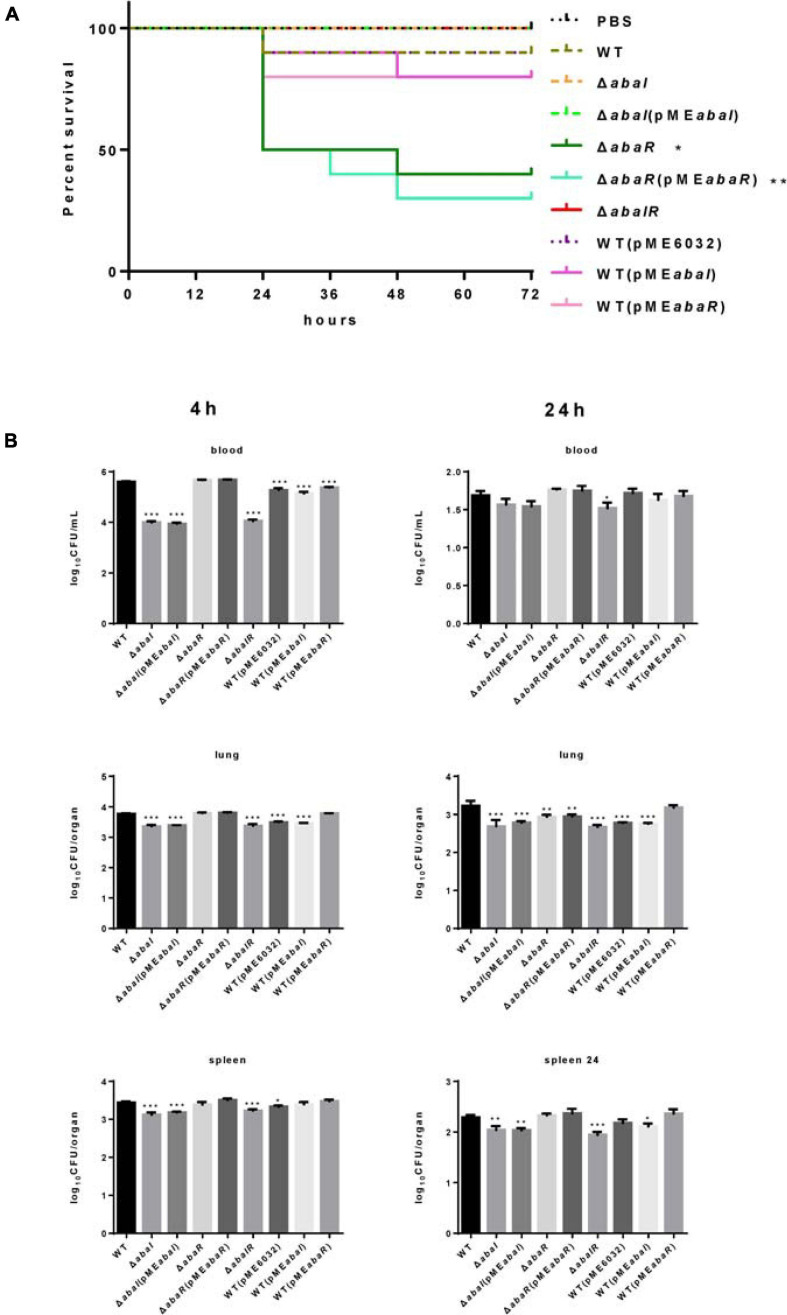
**(A)** Kaplan–Meier survival curve of mouse inoculated with strains at a dose of 1.2 × 108 CFUs (*n* = 10). Survival curves were compared using the log-rank (Mantel–Cox) test, *P < 0.05; **P < 0.005. **(B)** *A. baumannii* bacterial burdens in the lungs, spleen, and blood. Bacterial burdens in the blood and respective organs were determined by quantitative bacteriology at 4 and 24 h post inoculation. An unpaired *t*-test was used to validate the experimental data. *P < 0.05; **P < 0.005; ***P < 0.001.

Whether these QS genes of *A. baumannii* are important for virulence to a mammalian system is currently unknown. To evaluate the virulence of strains, we established a bacteremia model in mice by intraperitoneal injection of *A. baumannii*. In the experiments, we analyzed the survival of infected mice with this model and found that a dose of approximately 1.8 × 10^8^ CFU of Δ*abaI* and Δ*abaIR* mutants was unable to cause lethality, but only one mouse (10 per group) survived in the WT group, and all the mice in the Δ*abaR* group died (date not shown) after 48 h. Subsequently, we used a dose of approximately 1.2 × 10^8^ CFU of bacteria to infect 10 mice per group for survival studies. We observed that Δ*abaI* and Δ*abaIR* mutants were unable to cause lethality; WT exhibited a low fatality rate, but Δ*abaR* complemented with strain Δ*abaR*(pME*abaR*) can cause more deaths (Figure 6A). To explore the virulence of mutants in host resistance against *A. baumannii* infection, the blood, lungs, and spleens from BALB/C mice were collected at various time points after being injected with 1.2 × 10^8^ CFU of *A. baumannii*. We analyzed bacterial burdens in the blood, lung, and spleen of mice infected for 4, 24, and 72 h. As a result, the WT and Δ*abaR* mutant complemented with strain Δ*abaR*(pME*abaR*) resulted in an increase in bacterial counts, and Δ*abaI* and Δ*abaIR* mutants exhibited a remarkable reduction in the burden in blood, lung, and spleen at 4 h post inoculation. More specifically, as shown, Δ*abaI* and Δ*abaIR* mutants displayed a high serum clearance rate, resulting in a significant decrease in bacteremia at 4 h post inoculation. There were no differences at 24 h (Figure 6B). The bacteria were completely eliminated at 72 h (data not shown). These results indicated that deletion of *abaI* results in weaker toxicity in mouse models, while deletion of *abaR* results in enhanced virulence.

### Mutants Cause Differential Gene Expression in *A. baumannii* ATCC 17978

**FIGURE 7:**
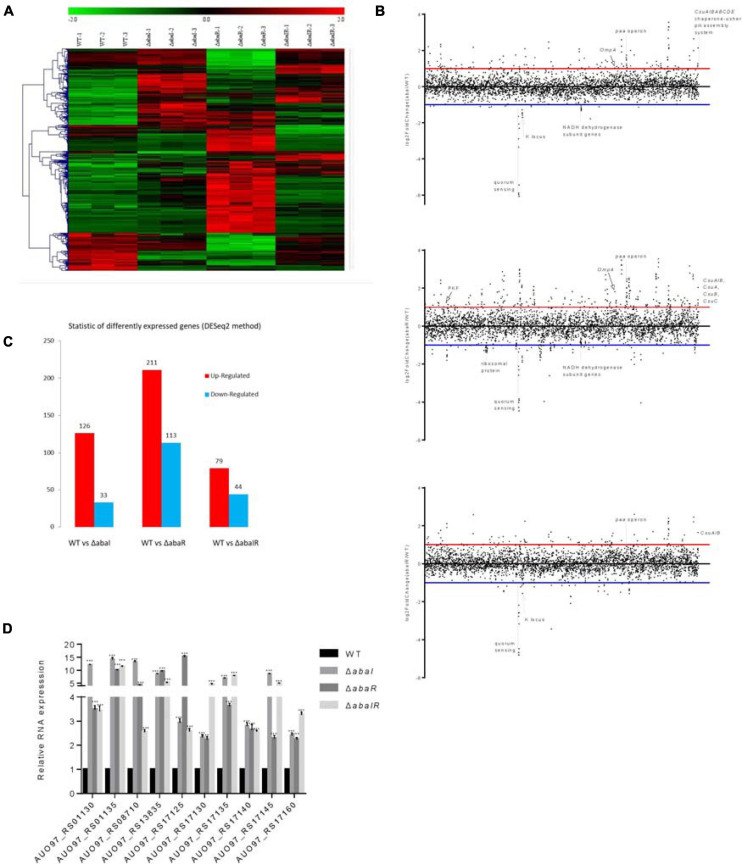
**(A)** Heat map representation and hierarchical clustering of gene expression changes. The *x*-coordinate represents the different experimental groups. Data represent differential gene expression profiles (PFKM) for genes listed along the ordinate. Red indicates increased expression, and green indicates decreased expression; color intensity indicates the magnitude of difference in expression according to the horizontal scale at the top. Black depicts genes with no significant difference in expression. **(B)** The genome-wide transcriptomic profile, genome-wide differential gene expression locus map, and plot of differential gene expression mutant compared with WT with respect to the gene locus tag number. **(C)** The vertical axis represents the number of significantly DEGs. The log2-fold change in expression for each gene meeting the study threshold (log2-fold change >1, false discovery rate < 0.001). The number of significantly DEGs can be divided into the number of significantly upregulated DEGs and the number of significantly downregulated DEGs. **(D)** Relative expression of 10 upregulated genes; results are presented relative to WT, which was normalized to 1. The results are expressed as mean ± SEM for at least three biological replicates. ***P < 0.001, *t*-test.

To identify transcriptional activity dependent on *abaI*/*abaR* function, RNA-seq analysis was performed on *abaI*/*abaR* mutants and the WT strain. A total of 463 genes were classified as differentially expressed in mutants relative to WT. Compared with that in WT, in the Δ*abaI* mutant, a total of 159 protein-coding genes (out of 3,848) were identified as differentially expressed by a log2-fold change greater than 1 or less than -1 (*P* ≤ 0.05) (126 with increased expression and 33 with decreased expression). Deletion of *abaR* had a larger impact on the transcriptome of strain ATCC 17978, with the differential expression of 324 genes (211 upregulated and 113 downregulated). The Δ*abaIR* mutant had a total of 123 DEGs (79 genes with increased expression and 44 with decreased expression) (Figures 7A–C and Supplementary Tables 1–3). These results revealed that partial changes in gene expression occur with changes in *abaI*/*abaR*. To validate our RNA-seq analysis, qRT-PCR was used to validate the 10 upregulated genes (Figure 7D). The results were consistent with the high-throughput sequencing data.

Given the different phenotypes of Δ*abaI*, Δ*abaR*, and Δ*abaIR* mutants, the RNA-seq analysis revealed a subset of the genes that was most highly activated or suppressed by the QS system, as shown in Figure 7B. We centralized our analysis on the gene subsets whose transcription was down in Δ*abaI*, Δ*abaR*, and Δ*abaIR* mutants; the QS gene (*abaI* AUO97_RS06645) and nearby locus (AUO97_RS06600–06630) showed strongly reduced transcription. One study found that in the *A. baumannii* M2 strain, QS mediated by the *abaI* is required for motility (Clemmer et al., 2011). We assessed the surface motility of the mutants, as shown in Figure 3. We observed that the WT strain exhibited a robust surface motility phenotype and that the mutants did not exhibit any signs of motility.

We mainly analyzed the gene expression of each group. Hemerythrin-like proteins have an effect on oxidation–reduction regulation and antibiotic resistance (Li et al., 2015). AUO97_RS11650 (hemerythrin) was downregulated in the Δ*abaI* mutant, and other antimicrobial resistance genes including AUO97_RS07490 (*mexK*), AUO97_RS07485 (*mexJ*), AUO97_RS07485 (efflux RND transporter periplasmic adaptor subunit), and AUO97_RS05665 (beta-lactamase domain protein) were downregulated. One gene, AUO97_RS16540 (*AdeA*/*Ade*I family multidrug efflux RND transporter periplasmic adaptor subunit) had a 1.2-fold increased expression in the Δ*abaR* mutant, while the expression of this gene was not changed in the Δ*abaIR* mutant. We then assessed the antimicrobial susceptibility of the mutants, as shown in Table 4, and found that the susceptibility of mutants toward a part of antimicrobials increased.

The *csu* operon is composed of six genes (*csuA*/*BABCDE*) and plays a central role in initial bacterial attachment and biofilm formation on abiotic surfaces (Tomaras et al., 2003). In the Δ*abaI* mutant, the *CsuA*/*BABCDE* chaperone–usher pili assembly system showed a high expression, except for the *CsuA* (AUO97_RS19210) gene. In the Δ*abaR* mutant, *CsuA/B* (AUO97_RS19215), *CsuA* (AUO97_RS19210), *CsuB* (AUO97_RS19205), and *CsuC* (AUO97_RS19200) were highly expressed, whereas in the Δ*abaIR* mutant, only *CsuA/B* (AUO97_RS19215) was highly expressed. *CsuA/B* is predicted to form part of the type I pili rod that was upregulated in all mutants (Figure 7B). Of particular interest is their regulator genes *bfmR*–*bfmS*, which did not change in all mutants. Biofilm formation was observed on the liquid surface of the Δ*abaI* and Δ*abaR* mutants (Figure 4A). A previous study found that *CsuC* and *CsuE* are required in the early steps of biofilm formation (Tomaras et al., 2003). In some *A. baumannii* strains, biofilms are not essential for virulence (Smith et al., 2007). Apart from the *csu* operon, other genes were controlled by QS, which may be related with biofilm formation. A1S_0644 (AUO97_RS08180), a hypothetical protein involved in biofilm formation, was repressed in the Δ*abaR* mutant.

Secreted bacterial proteins can mediate serum resistance; a secreted serine protease termed PKF is required for serum resistance and inhibits biofilm formation in *A. baumannii* (King et al., 2013). In the Δ*abaR* mutant, *PKF* (AUO97_RS01525) was highly expressed, and no difference was observed in the Δ*abaI* and Δ*abaIR* mutants (Figure 7B). We assessed the serum sensitivity test and the ability of mutants to form biofilms on the abiotic surface. As shown in Figures 4, 5A, WT and Δ*abaR* survived after incubation in serum, whereas Δ*abaI* and Δ*abaIR* were entirely killed after 1 h at 37°C. There was a remarkable decrease in the biofilm formation by the Δ*abaR* mutant. Apart from this gene, other genes that may be associated with serum resistance and biofilm formation may be regulated by QS.

**FIGURE 8:**
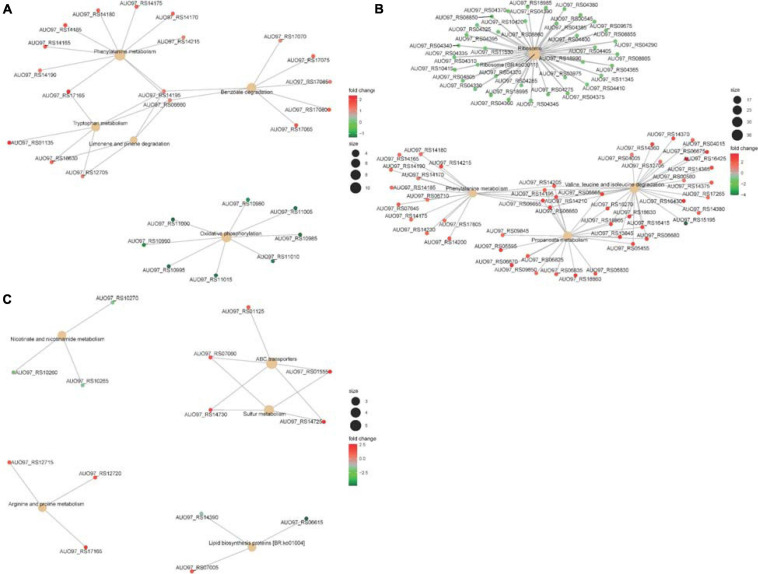
**(A)** The top five significantly enriched KEGG pathways of DEGs in Δ*abaI*. The size of the nodes shows the number of genes enriched in each pathway. The color gradient shows the log2-fold change of gene expression. **(B)** The top five significantly enriched KEGG pathways of DEGs in Δ*abaR*. The size of the nodes shows the number of genes enriched in each pathway. The color gradient shows the log2-fold change of gene expression. **(C)** The top five significantly enriched KEGG pathways of DEGs in Δ*abaIR*. The size of the nodes shows the number of genes enriched in each pathway. The color gradient shows the log2-fold change of gene expression.

NADH is mainly involved in material and energy metabolism in cells, which is transferring energy to ATP synthesis through oxidative phosphorylation on the mitochondrial inner membrane (Yanjun et al., 2019; Yu et al., 2019). In the respiratory chain of *A. baumannii*, there are 14 NADH-quinone oxidoreductase subunits involved in NADH dehydrogenase, which include *NuoA–NuoN*. In the Δ*abaI* mutant, a subset of genes including AUO97_RS10980–11015 (*NuoG*, *NuoH*, *NuoI*, *NuoJ*, *NouK*, *NuoL*, *NuoM*, and *NuoN*) was all downregulated (Figure 8A). In the Δ*abaR* mutant, AUO97_RS11000 (*NouK*) and AUO97_RS11015 (*NuoN*) were downregulated, and there was no change in the Δ*abaIR* mutant. One study found that these genes are essential in affecting growth in the LB medium (Wang et al., 2014). We then assessed the growth characteristics and morphology of the mutants, as shown in Figures 1A,B; the Δ*abaI* mutant showed slightly slowed growth at the logarithmic phase, the cytoplasm of the Δ*abaI* mutant appeared to be transparent, and the cytoplasmic density of the Δ*abaI* mutant is relatively low. These genes play a significant role in mediating cell growth and energy metabolism in *A. baumannii*. Apart from NADH dehydrogenase, other genes that may be associated with cell growth and energy metabolism may be regulated by QS. In the Δ*abaI* mutant, a subset of genes involved in benzoate degradation (AUO97_RS17065–17085) and tryptophan metabolism and limonene and pinene degradation was upregulated; this strain may utilize the beta-ketoadipate pathway and tryptophan for energy supply (Figure 8A).

The phenylacetic acid (PAA) catabolic pathway encoded by the *paa* operon is a key route in the catabolism of the Krebs cycle, and this pathway is thought to contribute to bacterial virulence (Teufel et al., 2010; Cerqueira et al., 2014). The cluster is composed of 15 coding sequences (*paaZ*, *paaA*, *paaB*, *paaC*, *paaD*, *paaE*, *paaF*, *paaG*, *paaH*, *paaJ*, *paaK1*, *paaK2*, *paaX*, *paaY*, and *paaI*). In the Δ*abaR* mutant, the *paa* operon was highly expressed (Figure 8B). In the Δ*abaI* mutant, AUO97_RS14165 (*paaZ*), AUO97_RS14170 (*paaA*), AUO97_RS14175 (*paaB*), AUO97_RS14180 (*paaC*), AUO97_RS14185 (*paaD*), AUO97_RS14190 (*paaE*), AUO97_RS14195 (*paaF*), and AUO97_RS14215 (*paaK1*) were highly expressed (Figure 8A), and the expression of this operon was not changed in the Δ*abaIR* mutant.

Branched-chain amino acids (BCAAs), including leucine (Leu), isoleucine (Ile), and valine (Val), are vital to both growth and virulence in bacteria (Kaiser et al., 2016; Kim G.L. et al., 2017). In the Δ*abaR* mutant, the Val, Leu, and Ile degradation pathways were upregulated (Figure 8B). The BCAAs serve as precursors for branched-chain fatty acids (BCFAs), which are predominant membrane fatty acids; the BCAAs are key co-regulators of virulence factors.

Propionate is one of the most abundant short-chain fatty acids (SCFAs). In bacteria, propionate catabolism plays an important role in virulence (Dolan et al., 2018). In the Δ*abaR* mutant, there are 27 genes involved in the propanoate metabolism pathway that were upregulated (Figure 8B). In Δ*abaIR* mutant, Nicotinate and nicotinamide metabolism and Lipid biosynthesis proteins are reduced. Sulfur metabolism and Arginine and proline metabolism were enhanced, which may contribute to the virulence of the strain (Figure 8C). We assessed the virulence of the mutant in *G. mellonella*. As shown in Figure 5B, the Δ*abaIR* mutant enhances slightly more virulent than Δ*abaI*. In the Δ*abaI* mutant, AUO97_RS14195, AUO97_RS16430, AUO97_RS06660, AUO97_RS18630, and AUO97_RS12705 involved in the propanoate metabolism pathway were upregulated. In the Δ*abaIR* mutant, AUO97_RS14380, AUO97_RS14375, and AUO97_RS14370 involved in the propanoate metabolism pathway were upregulated.

**FIGURE 9:**
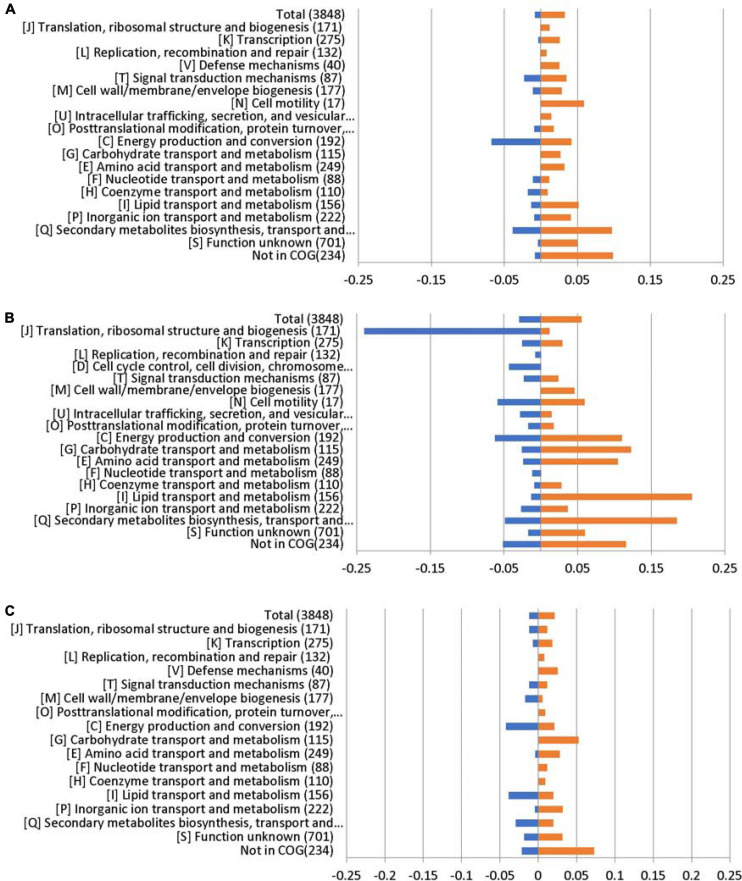
RNA sequencing results displayed by COG enrichment for differentially regulated genes. COG enrichment analysis was performed by dividing the percentage of genes upregulated or downregulated for each category by the percentage of genes in that category across the whole genome. **(A)** Δ*abaI*; **(B)** Δ*abaR*; and **(C)** Δ*abaIR*.

Lipids play an important role in both the physiology and pathophysiology of living systems (Pereira-Dutra et al., 2019). Membrane phospholipids play a key role in the defense against antimicrobials, including host fatty acids (Eijkelkamp et al., 2018; Beavers et al., 2019; Jiang et al., 2019). The COG enrichment analysis indicated that a large amount of genes with predicted functions in [I] lipid transport and metabolism (21%) was upregulated in the Δ*abaR* mutant (Figure 9). PAAs, BCAAs, SCFAs, and lipid transport and metabolism may play a protective role against the host and serum.

The K locus regulates the production of complex polysaccharides to protect against killing by host serum and enhance virulence in animal models of infection (Geisinger and Isberg, 2015). In the K locus (O-glycosylation and wzy-dependent capsule synthesis locus) (AUO97_RS06870–06965), the gene AUO97_RS06875 (UDP-glucose 4-epimerase GalE) showed weakened transcription in the Δ*abaI* and Δ*abaIR* mutants by a 1.7-fold decrease and a 1.6-fold decrease, respectively, while there was no change in the Δ*abaR* mutant (Figure 7B).

AUO97_RS13365 (OMPA family protein), a naturally glycosylated protein in *A. baumannii* ATCC 17978, was expressed with a 1.8-fold increase in Δ*abaR* strain, and no difference in Δ*abaI* and Δ*abaIR* mutants was observed (Figure 7B).

Ribosomal proteins (RPs) are well known for their role in mediating protein synthesis and maintaining the stability of the ribosomal complex, which includes small and large subunits. There were 36 genes encoding for RPs that exhibited reduced expression in the Δ*abaR* mutant strain (Figure 8B). Twenty-three (L1–L6, L9–L13, L15–L20, L22–L23, L25, L28, L29, and L35) of them were associated with the large subunit while the remaining 13 (S2–S8, S10, S11, S14, and S17–S19) were associated with the small subunit. This change may enable *A. baumannii* to “fine-tune” their proteomes to regulate the pathogenicity of bacteria. There was no change in Δ*abaI* and Δ*abaIR* mutants. We assessed the virulence of the mutant in *G. mellonella* and mouse and serum sensitivity tests. As shown in Figures 5A,B, 6, the Δ*abaR* mutant enhances more virulence and serum resistance, while Δ*abaI* and Δ*abaIR* mutants markedly attenuated the virulence of *A. baumannii*. The selected genes (*paaG*, *paaH*, *paaJ*, *paaK2*, *paaX*, *paaY*, and *paaI*) encode proteins in the PAA catabolic pathway and BCAAs, and the capsule synthesis loci *GalE* and *OmpA* may contribute to virulence.

### COG Annotation

To link transcriptional reprogramming by *abaI*/*abaR* to function, DEGs were categorized into COGs (Tatusov et al., 2000; Huerta-Cepas et al., 2017). The COG enrichment analysis identified that 7% of the genes belonging to the COG category [C] energy production and conversion were significantly repressed and that 10% of the genes were associated with the COG category [Q] secondary metabolites biosynthesis, transport, and catabolism were highly regulated in the Δ*abaI* mutant. We observed a high proportion of genes with increased expression in the Δ*abaR* mutant that encoded proteins involved in [I] lipid transport and metabolism (21%); [Q] secondary metabolites biosynthesis, transport, and catabolism (18%); [G] carbohydrate transport and metabolism (12%); [C] energy production and conversion (11%); and [E] amino acid transport and metabolism (10%), while observing a strong decrease in [J] translation, ribosomal structure, and biogenesis (24%). In the Δ*abaIR* mutant, 4% of the genes belonging to the COG category [C] energy production and conversion and [I] lipid transport and metabolism were downregulated, while 5% of the genes belonging to the COG category [G] carbohydrate transport and metabolism were upregulated (Figure 9).

### GO and KEGG Pathway Enrichment Analyses of DEGs

**FIGURE 10:**
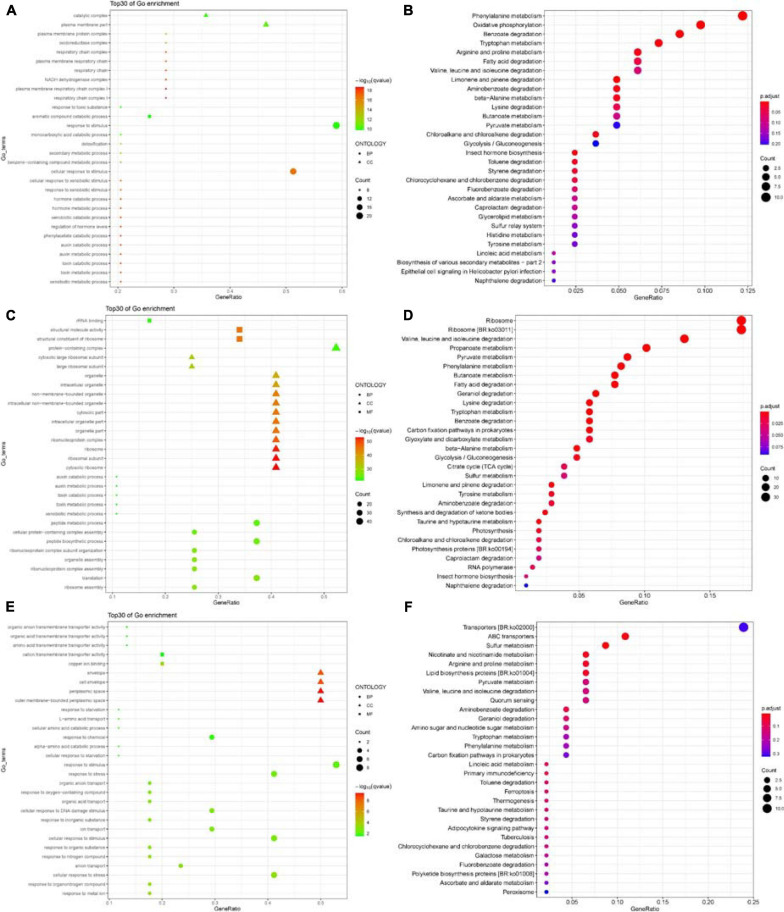
**(A)** Top 30 GO enrichment terms of DEGs in Δ*abaI*. The shape of the point indicates the different ontologies. The enrichment *q*-value of each GO term was normalized as negative log10(*q*-value) and is shown as a color gradient. The number of genes enriched in each GO term is represented by the size of the points. **(B)** The significantly enriched KEGG pathway of DEGs in Δ*abaI*. The enrichment *P*-adjusted value of each pathway was shown as a color gradient. GeneRatio, number of DEGs/total number of genes in this KEGG pathway. The number of genes enriched in each pathway is represented by the size of the points. **(C)** Top 30 GO enrichment terms of DEGs in Δ*abaR*. The shape of the point indicates the different ontologies. The enrichment *q*-value of each GO term was normalized as negative log10(*q*-value) and is shown as a color gradient. The number of genes enriched in each GO term is represented by the size of the points. **(D)** The significantly enriched KEGG pathway of DEGs in Δ*abaR*. The enrichment *P*-adjusted value of each pathway was shown as a color gradient. GeneRatio, number of DEGs/total number of genes in this KEGG pathway. The number of genes enriched in each pathway is represented by the size of the points. **(E)** Top 30 GO enrichment terms of DEGs in Δ*abaIR*. The shape of the point indicates the different ontologies. The enrichment *q*-value of each GO term was normalized as negative log10(*q*-value) and is shown as a color gradient. The number of genes enriched in each GO term is represented by the size of the points. **(F)** The significantly enriched KEGG pathway of DEGs in Δ*abaIR*. The enrichment *P*-adjusted value of each pathway was shown as a color gradient. GeneRatio, number of DEGs/total number of genes in this KEGG pathway. The number of genes enriched in each pathway is represented by the size of the points.

The functions of the DEGs were annotated and classified based on GO and KEGG pathway enrichment analyses. The top 30 significant GO terms were divided into two major categories – biological process and cellular component in the Δ*abaI* mutant – as shown in Figure 10A. Among the biological processes, DEGs were distributed to the xenobiotic metabolic process, toxin metabolic/catabolic process, auxin metabolic/catabolic process, phenylacetate catabolic process, and so on. In the cellular component field, DEGs belonged to respiratory chain complex I, plasma membrane respiratory chain complex I, NADH dehydrogenase complex, and respiratory chain. The top 30 significant GO terms were divided into three major categories – biological process, cellular component, and molecular function in Δ*abaR* mutant – as shown in Figure 10C. Among the biological processes, DEGs were distributed to ribosome assembly, translation, ribonucleoprotein complex assembly, organelle assembly, ribonucleoprotein complex subunit organization, and peptide biosynthetic process. In the cellular component field, DEGs belonged to cytosolic ribosome, ribosome, ribonucleoprotein complex, organelle part, and cytosolic part. In molecular function, DEGs were responsible for the structural constituent of ribosome, structural molecule activity, and rRNA binding. The top 30 significant GO terms were divided into three major categories – biological process, cellular component, and molecular function in the Δ*abaIR* mutant – as shown in Figure 10E. Among the biological processes, DEGs were distributed to response to metal ion, response to organonitrogen compound, cellular response to stress, and anion transport. In the cellular component field, DEGs belonged to the outer membrane-bounded periplasmic space, periplasmic space, cell envelope, and envelope. In molecular function, DEGs were responsible for copper ion binding and cation/amino acid/organic acid/organic anion transmembrane transporter activity.

Kyoto Encyclopedia of Genes and Genomes pathway enrichment suggested that DEGs of the Δ*abaI* mutant were significantly enriched in the pathways related to phenylalanine metabolism, oxidative phosphorylation, benzoate degradation, tryptophan metabolism, arginine and proline metabolism, fatty acid degradation, and so on (Figure 10B). DEGs of the Δ*abaR* mutant were mainly involved in the pathways related to ribosome; Val, Leu, and Ile degradation; propanoate metabolism; pyruvate metabolism; phenylalanine metabolism; fatty acid degradation; and so on (Figure 10D). DEGs of the Δ*abaIR* mutant were mainly involved in the pathways related to ABC transporters, sulfur metabolism, nicotinate and nicotinamide metabolism, and so on (Figure 10F).

## Discussion

*Acinetobacter baumannii* has become a very important hospital-acquired pathogen. Bacterial virulence is the prime determinant for the deterioration of an infected patient’s health. QS is a cell-to-cell communication system utilized by bacteria to promote collective behaviors. Many bacteria use QS to control virulence.

Our lab’s previous research found that the *abaI*/*abaR* QS system was widely distributed among the *A. baumannii* clinical isolates, was necessary for surface-related motility, and was significantly correlated with drug resistance and virulence to *G. mellonella* (Tang et al., 2020). In the present study, we focused on detecting the role of the *abaI*/*abaR* QS system in the virulence of *A. baumannii* ATCC 17978. The mutant lacking *abaI* is believed to be less virulent than the WT strain. In contrast, the *abaR* mutants were significantly more pathogenic than the WT strain. This result was confirmed in our study by injection of *G. mellonella* larvae and a mouse model of infection and serum killing test. Our transcriptomic analysis results revealed that deletion of *abaI* leads to the significant repression of energy production and conversion genes. The connection between energy metabolism and virulence has been reported in a multitude of bacteria. In *V. cholerae*, the expression of virulence regulatory protein *ToxT* is affected by the NADH via respiration activity (Minato et al., 2013). In *Pseudomonas savastanoi*, *RhpR* directly regulates multiple metabolic pathways and phosphorylation to specifically control virulence (Xie et al., 2019). Therefore, *abaI* may indirectly control bacterial virulence by inducing the differential expression of some key genes involved in NADH dehydrogenase in the respiratory chain. Deletion of *abaR* enhances more cytotoxicity and immune evasion. RNA-seq analysis indicated that deletion of *abaR* leads to the significant overexpression of lipid transport and metabolism, carbohydrate transport and metabolism, and amino acid transport and metabolism genes. Lipid metabolism plays a key role in the pathogenicity of some intracellular bacteria (Rameshwaram et al., 2018). It has been observed that lipids are the main carbon and energy source of *Mycobacterium tuberculosis*, which switches from carbohydrate utilization to the fatty acid utilization pathway for the establishment of a successful infection (Mukhopadhyay et al., 2012). *A. baumannii* is a ubiquitous, facultative intracellular bacterial pathogen (Tang et al., 2018). The Δ*abaR* mutant may enhance lipid transport and metabolism to play a protective role against the host. The Δ*abaIR* mutant was slightly more pathogenic than the Δ*abaI* mutants but less pathogenic than the Δ*abaR* mutants. This result was verified in our study by injection of bacteria into *G. mellonella* larvae.

Virulence through the intermediated phenylacetate catabolism pathway has been found in *A. baumannii*, and deletion of *paaE* attenuated *A. baumannii* virulence in the mouse septicemia model (Cerqueira et al., 2014). In *Burkholderia cenocepacia*, *paaA* and *paaE* insertional mutants showed reduced virulence, and interruption of *paaZ* and *paaF* slightly increased virulence in the *Caenorhabditis elegans* model of infection (Law et al., 2008). Therefore, deletion of the *abaR* gene may indirectly enhance bacterial virulence via triggering the differential expression of a lot of key genes involved in the phenylacetate catabolism pathway. The selected genes (*paaG*, *paaH*, *paaJ*, *paaK2*, *paaX*, *paaY*, and *paaI*) encode proteins in the PAA catabolic pathway that may contribute to virulence. *abaI* is a protein that synthesizes AHLs, and *abaR* is a LuxR homolog transcription factor/receptor for AHLs (Stacy et al., 2012). In this study, only the WT strain was observed to produce AHLs based on *A. tumefaciens* KYC55 reporter strains, while the green pigment was not observed in Δ*abaI*, Δ*abaR*, and Δ*abaIR* mutants. No purple pigment was observed in all strains based on *C. violaceum* CV026. In a previous study, the absence of purple pigment may be attributed to the low rate of production of short-chain homoserine lactone and fast degradation in the strains (Erdonmez et al., 2017). Our transcriptomic analysis results revealed that deletion of *abaR* leads to the significant repression of *abaI*. Therefore, *abaR* may be a repressor such that repression is relieved when AHLs are bound. The results suggest that *abaR* generally represses its regulon of genes until it binds AHLs. When AHLs are bound, then repression is relieved. This may explain why deletion of *abaI* is substantially different from deletion of *abaR*. When *abaI* is mutated, then *abaR* still represses the expression of many genes, such as those associated with virulence. When both genes were knocked out, the virulence of the strain was moderate to low level.

The present research will help advance the functional genomic analysis of the QS system in *A. baumannii* and provides a new insight into *abaI*/*abaR* QS system effects on pathogenicity in *A. baumannii*. We propose that targeting the AHL synthase enzyme *abaI* could provide an effective strategy for attenuating virulence. On the contrary, interdicting the AI synthase receptor *abaR* elicits unpredictable consequences, which may lead to enhanced bacterial virulence.

## Data Availability Statement

The datasets presented in this study can be found in online repositories. The names of the repository/repositories and accession number(s) can be found in the article/Supplementary Material.

## Ethics Statement

The animal study was reviewed and approved by Experimental Animal Center, Jilin University. Written informed consent was obtained from the owners for the participation of their animals in this study.

## Author Contributions

ZN and FL designed the experiments and revised the manuscript. XS performed the experiments, analyzed the data and wrote the manuscript. JT and YD performed the experiments. XW interpreted the data. All authors contributed to the article and approved the submitted version.

## Conflict of Interest

The authors declare that the research was conducted in the absence of any commercial or financial relationships that could be construed as a potential conflict of interest.

## Footnotes

1 http://www.basel.declaration.org

## References

[B1] Amin I. M. Richmond G. E. Sen P. Koh T. H. Piddock L. J. Chua K. L. (2013). A method for generating marker-less gene deletions in multidrug-resistant *Acinetobacter baumannii*. *BMC Microbiol.* 13:158. 10.1186/1471-2180-13-158 23848834 PMC3717142

[B2] Beavers W. N. Monteith A. J. Amarnath V. Mernaugh R. L. Roberts L. J. Chazin W. J. (2019). Arachidonic acid kills staphylococcus aureus through a lipid peroxidation mechanism. *mBio* 1 10. 10.1128/mBio.01333-19 PMC6775451 31575763

[B3] Bhargava N. Sharma P. Capalash N. (2010). Quorum sensing in Acinetobacter: an emerging pathogen. *Crit. Rev. Microbiol.* 36 349–360. 10.3109/1040841x.2010.512269 20846031

[B4] Bhargava N. Singh S. P. Sharma A. Sharma P. Capalash N. (2015). Attenuation of quorum sensing-mediated virulence of *Acinetobacter baumannii* by Glycyrrhiza glabra flavonoids. *Future Microbiol.* 10 1953–1968. 10.2217/fmb.15.107 26582430

[B5] Cerqueira G. M. Kostoulias X. Khoo C. Aibinu I. Qu Y. Traven A. (2014). A global virulence regulator in *Acinetobacter baumannii* and its control of the phenylacetic acid catabolic pathway. *J. Infect. Dis.* 1 46–55. 10.1093/infdis/jiu024 24431277

[B6] Clemmer K. M. Bonomo R. A. Rather P. N. (2011). Genetic analysis of surface motility in *Acinetobacter baumannii*. *Microbiology* 157 2534–2544. 10.1099/mic.0.049791-0 21700662 PMC3352170

[B7] Dolan S. K. Wijaya A. Geddis S. M. Spring D. R. Silva-Rocha R. Welch M. (2018). Loving the poison: the methylcitrate cycle and bacterial pathogenesis. *Microbiology* 164 251–259. 10.1099/mic.0.000604 29458664

[B8] Dou Y. Song F. Guo F. Zhou Z. Zhu C. Xiang J. (2017). *Acinetobacter baumannii* quorum-sensing signalling molecule induces the expression of drug-resistance genes. *Mol. Med. Rep.* 15 4061–4068. 10.3892/mmr.2017.6528 28487993 PMC5436197

[B9] Eijkelkamp B. A. Begg S. L. Pederick V. G. Trapetti C. Gregory M. K. Whittall J. J. (2018). Arachidonic acid stress impacts pneumococcal fatty acid homeostasis. *Front. Microbiol.* 9:813. 10.3389/fmicb.2018.00813 29867785 PMC5958418

[B10] Erdonmez D. Rad A. Y. Aksoz N. (2017). Quorum sensing molecules production by nosocomial and soil isolates *Acinetobacter baumannii*. *Arch. Microbiol.* 199 1325–1334. 10.1007/s00203-017-1408-8 28688010

[B11] Fernandez-Garcia L. Ambroa A. Blasco L. Bleriot I. Lopez M. Alvarez-Marin R. (2018). Relationship between the quorum network (sensing/quenching) and clinical features of pneumonia and bacteraemia caused by a. baumannii. *Front. Microbiol.* 9:3105. 10.3389/fmicb.2018.03105 30619184 PMC6304438

[B12] Galperin M. Y. Makarova K. S. Wolf Y. I. Koonin E. V. (2015). Expanded microbial genome coverage and improved protein family annotation in the COG database. *Nucleic Acids Res.* 43 D261–D269. 25428365 10.1093/nar/gku1223 PMC4383993

[B13] Geisinger E. Isberg R. R. (2015). Antibiotic modulation of capsular exopolysaccharide and virulence in *Acinetobacter baumannii*. *PLoS Pathog.* 11:e1004691. 10.1371/journal.ppat.1004691 25679516 PMC4334535

[B14] Harding C. M. Hennon S. W. Feldman M. F. (2018). Uncovering the mechanisms of *Acinetobacter baumannii* virulence. *Nat. Rev. Microbiol.* 16 91–102. 10.1038/nrmicro.2017.148 29249812 PMC6571207

[B15] Harris G. Kuo Lee R. Lam C. K. Kanzaki G. Patel G. B. Xu H. H. (2013). A mouse model of *Acinetobacter baumannii*-associated pneumonia using a clinically isolated hypervirulent strain. *Antimicrob. Agents. Chemother.* 57 3601–3613. 10.1128/aac.00944-13 23689726 PMC3719758

[B16] Heeb S. Itoh Y. Nishijyo T. Schnider U. Keel C. Wade J. (2000). Small, stable shuttle vectors based on the minimal pVS1 replicon for use in gram-negative, plant-associated bacteria. *Mol. Plant Microbe. Interact.* 13 232–237. 10.1094/mpmi.2000.13.2.232 10659714

[B17] Herzog R. Peschek N. Frohlich K. S. Schumacher K. Papenfort K. (2019). Three autoinducer molecules act in concert to control virulence gene expression in *Vibrio cholerae*. *Nucleic Acids Res.* 8 3171–3183. 10.1093/nar/gky1320 30649554 PMC6451090

[B18] Howe E. A. Sinha R. Schlauch D. Quackenbush J. (2011). RNA-Seq analysis in MeV. *Bioinformatics* 15 3209–3210. 10.1093/bioinformatics/btr490 21976420 PMC3208390

[B19] Huerta-Cepas J. Forslund K. Coelho L. P. Szklarczyk D. Jensen L. J. von Mering C. (2017). Fast genome-wide functional annotation through orthology assignment by eggNOG-mapper. *Mol. Biol. Evol.* 1 2115–2122. 10.1093/molbev/msx148 28460117 PMC5850834

[B20] Jiang J. H. Hassan K. A. Begg S. L. Rupasinghe T. W. T. Naidu V. Pederick V. G. (2019). Identification of novel *Acinetobacter baumannii* host fatty acid stress adaptation strategies. *mBio* 5:10. 10.1128/mBio.02056-18 PMC6428749 30723122

[B21] Kaiser J. C. Sen S. Sinha A. Wilkinson B. J. Heinrichs D. E. (2016). The role of two branched-chain amino acid transporters in Staphylococcus aureus growth, membrane fatty acid composition and virulence. *Mol. Microbiol.* 102 850–864. 10.1111/mmi.13495 27589208 PMC6225994

[B22] Kaplan J. B. Izano E. A. Gopal P. Karwacki M. T. Kim S. Bose J. L. (2012). Low levels of beta-lactam antibiotics induce extracellular DNA release and biofilm formation in Staphylococcus aureus. *mBio* 3 e198–e112. 10.1128/mBio.00198-12 PMC3419523 22851659

[B23] Kim G. L. Lee S. Luong T. T. Nguyen C. T. Park S. S. Pyo S. (2017). Effect of decreased BCAA synthesis through disruption of ilvC gene on the virulence of Streptococcus pneumoniae. *Arch. Pharm. Res.* 40 921–932. 10.1007/s12272-017-0931-0 28735462

[B24] Kim M. K. Zhao A. Wang A. Brown Z. Z. Muir T. W. Stone H. A. (2017). Surface-attached molecules control Staphylococcus aureus quorum sensing and biofilm development. *Nat. Microbiol.* 22:17080. 10.1038/nmicrobiol.2017.80 PMC5526357 28530651

[B25] King L. B. Pangburn M. K. McDaniel L. S. (2013). Serine protease PKF of *Acinetobacter baumannii* results in serum resistance and suppression of biofilm formation. *J. Infect. Dis.* 207 1128–1134. 10.1093/infdis/jis939 23303803

[B26] Lade H. Paul D. Kweon J. H. (2014). Isolation and molecular characterization of biofouling bacteria and profiling of quorum sensing signal molecules from membrane bioreactor activated sludge. *Int. J. Mol. Sci.* 4 2255–2273. 10.3390/ijms15022255 24499972 PMC3958849

[B27] Law R. J. Hamlin J. N. Sivro A. McCorrister S. J. Cardama G. A. Cardona S. T. (2008). A functional phenylacetic acid catabolic pathway is required for full pathogenicity of Burkholderia cenocepacia in the Caenorhabditis elegans host model. *J. Bacteriol.* 190 7209–7218. 10.1128/jb.00481-08 18776009 PMC2580687

[B28] Li H. Li X. Song C. Zhang Y. Wang Z. Liu Z. (2017). Autoinducer-2 facilitates *Pseudomonas aeruginosa* PAO1 pathogenicity in vitro and in vivo. *Front. Microbiol.* 8:1944. 10.3389/fmicb.2017.01944 29089927 PMC5651085

[B29] Li X. Li J. Hu X. Huang L. Xiao J. Chan J. (2015). Differential roles of the hemerythrin-like proteins of Mycobacterium smegmatis in hydrogen peroxide and erythromycin susceptibility. *Sci. Rep.* 26 16130. 10.1038/srep16130 PMC4660385 26607739

[B30] Liu D. Liu Z. S. Hu P. Cai L. Fu B. Q. Li Y. S. (2016). Characterization of surface antigen protein 1 (SurA1) from *Acinetobacter baumannii* and its role in virulence and fitness. *Vet. Microbiol.* 15 126–138. 10.1016/j.vetmic.2016.02.018 27016767

[B31] Miller M. B. Bassler B. L. (2001). Quorum sensing in bacteria. *Ann. Rev. Microbiol.* 55 165–199. 11544353 10.1146/annurev.micro.55.1.165

[B32] Minato Y. Fassio S. R. Wolfe A. J. Hase C. C. (2013). Central metabolism controls transcription of a virulence gene regulator in *Vibrio cholerae*. *Microbiology* 159 792–802. 10.1099/mic.0.064865-0 23429745 PMC3709826

[B33] Mukherjee S. Moustafa D. A. Stergioula V. Smith C. D. Goldberg J. B. Bassler B. L. (2018). The PqsE and RhlR proteins are an autoinducer synthase-receptor pair that control virulence and biofilm development in *Pseudomonas aeruginosa*. *Proc. Natl. Acad. Sci. U.S.A.* 2 E9411–E9418. 10.1073/pnas.1814023115 PMC6176596 30224496

[B34] Mukhopadhyay S. Nair S. Ghosh S. (2012). Pathogenesis in tuberculosis: transcriptomic approaches to unraveling virulence mechanisms and finding new drug targets. *FEMS Microbiol. Rev.* 36 463–485. 10.1111/j.1574-6976.2011.00302.x 22092372

[B35] Niu C. Clemmer K. M. Bonomo R. A. Rather P. N. (2008). Isolation and characterization of an autoinducer synthase from *Acinetobacter baumannii*. *J. Bacteriol.* 190 3386–3392. 10.1128/jb.01929-07 18281398 PMC2347373

[B36] Peleg A. Y. Jara S. Monga D. Eliopoulos G. M. Moellering R. C. Jr. Mylonakis E. (2009). Galleria mellonella as a model system to study *Acinetobacter baumannii* pathogenesis and therapeutics. *Antimicrob. Agents Chemother.* 53 2605–2609. 10.1128/aac.01533-08 19332683 PMC2687231

[B37] Pereira-Dutra F. S. Teixeira L. de Souza Costa M. F. Bozza P. T. (2019). Fat, fight, and beyond: the multiple roles of lipid droplets in infections and inflammation. *J. Leukoc. Biol.* 106 563–580. 10.1002/jlb.4mr0119-035r 31121077

[B38] Rameshwaram N. R. Singh P. Ghosh S. Mukhopadhyay S. (2018). Lipid metabolism and intracellular bacterial virulence: key to next-generation therapeutics. *Future Microbiol.* 13 1301–1328. 10.2217/fmb-2018-0013 30256124

[B39] Rutherford S. T. Bassler B. L. (2012). Bacterial quorum sensing: its role in virulence and possibilities for its control. *Cold Spring Harb. Perspect. Med.* 1:2. 10.1101/cshperspect.a012427 PMC3543102 23125205

[B40] Smani Y. Dominguez-Herrera J. Pachon J. (2013). Association of the outer membrane protein Omp33 with fitness and virulence of *Acinetobacter baumannii*. *J. Infect. Dis.* 15 1561–1570. 10.1093/infdis/jit386 23908480

[B41] Smith M. G. Gianoulis T. A. Pukatzki S. Mekalanos J. J. Ornston L. N. Gerstein M. (2007). New insights into *Acinetobacter baumannii* pathogenesis revealed by high-density pyrosequencing and transposon mutagenesis. *Genes Dev.* 1 601–614. 10.1101/gad.1510307 17344419 PMC1820901

[B42] Stacy D. M. Welsh M. A. Rather P. N. Blackwell H. E. (2012). Attenuation of quorum sensing in the pathogen *Acinetobacter baumannii* using non-native N-Acyl homoserine lactones. *ACS Chem. Biol.* 19 1719–1728. 10.1021/cb300351x PMC3477293 22853441

[B43] Tang J. Chen Y. Wang X. Ding Y. Sun X. Ni Z. (2020). Contribution of the AbaI/AbaR quorum sensing system to resistance and virulence of *Acinetobacter baumannii* clinical strains. *Infec. Drug Resist.* 13 4273–4281. 10.2147/idr.s276970 33262621 PMC7699449

[B44] Tang J. Zhu H. Cai L. Tang T. Tang J. Sun Y. (2018). Postoperative infection caused by *Acinetobacter baumannii* misdiagnosed as a free-living amoeba species in a humeral head hemiarthroplasty patient: a case report. *Infect. Dis. Poverty.* 31:33. 10.1186/s40249-018-0408-5 PMC5890356 29631621

[B45] Tatusov R. L. Galperin M. Y. Natale D. A. Koonin E. V. (2000). The COG database: a tool for genome-scale analysis of protein functions and evolution. *Nucleic Acids Res.* 1 33–36. 10.1093/nar/28.1.33 10592175 PMC102395

[B46] Teufel R. Mascaraque V. Ismail W. Voss M. Perera J. Eisenreich W. (2010). Bacterial phenylalanine and phenylacetate catabolic pathway revealed. *Proc. Nat. Acad. Sci. U.S.A.* 10 14390–14395. 10.1073/pnas.1005399107 20660314 PMC2922514

[B47] Tomaras A. P. Dorsey C. W. Edelmann R. E. Actis L. A. (2003). Attachment to and biofilm formation on abiotic surfaces by *Acinetobacter baumannii*: involvement of a novel chaperone-usher pili assembly system. *Microbiology* 149 3473–3484. 10.1099/mic.0.26541-0 14663080

[B48] Vila-Farres X. Parra-Millan R. Sanchez-Encinales V. Varese M. Ayerbe-Algaba R. Bayo N. (2017). Combating virulence of gram-negative bacilli by ompa inhibition. *Sci. Rep.* 31:14683. 10.1038/s41598-017-14972-y PMC5666006 29089624

[B49] Wang N. Ozer E. A. Mandel M. J. Hauser A. R. (2014). Genome-wide identification of *Acinetobacter baumannii* genes necessary for persistence in the lung. *mBio* 3 e1163–e1114. 10.1128/mBio.01163-14 PMC4049102 24895306

[B50] Wong D. Nielsen T. B. Bonomo R. A. Pantapalangkoor P. Luna B. Spellberg B. (2017). Clinical and pathophysiological overview of acinetobacter infections: a century of challenges. *Clin. Microbiol. Rev.* 30 409–447. 10.1128/cmr.00058-16 27974412 PMC5217799

[B51] Xie Y. Shao X. Zhang Y. Liu J. Wang T. Zhang W. (2019). *Pseudomonas* savastanoi two-component system rhprs switches between virulence and metabolism by tuning phosphorylation state and sensing nutritional conditions. *mBio* 19:10. 10.1128/mBio.02838-18 PMC6426608 30890603

[B52] Yanjun S. Shuixiu L. Yajing Z. Yishan Z. Yan L. Yuanying J. (2019). ADH1 promotes Candida albicans pathogenicity by stimulating oxidative phosphorylation. *Int. J. Med. Microbiol.* 309:151330. 10.1016/j.ijmm.2019.151330 31471070

[B53] Yu G. Wang L. G. Han Y. He Q. Y. (2012). clusterProfiler: an R package for comparing biological themes among gene clusters. *OMICS* 16 284–287. 10.1089/omi.2011.0118 22455463 PMC3339379

[B54] Yu Z. Zhu Y. Fu J. Qiu J. Yin J. (2019). Enhanced NADH metabolism involves colistin-induced killing of bacillus subtilis and paenibacillus polymyxa. *Molecules* 22 24. 10.3390/molecules24030387 PMC6384706 30678237

[B55] Zuo J. Yin H. Hu J. Miao J. Chen Z. Qi K. (2019). Lsr operon is associated with AI-2 transfer and pathogenicity in avian pathogenic *Escherichia coli*. *Vet. Res.* 12:109. 10.1186/s13567-019-0725-0 PMC6909531 31831050

