# Supplementary material for: The abaI/abaR Quorum Sensing System Effects on Pathogenicity in Acinetobacter baumannii
Source: Front Microbiol. 2021 Jul 12;12:679241. doi: 10.3389/fmicb.2021.679241 (PMC8312687; doi:10.3389/fmicb.2021.679241)
Supplement: Supplementary file 1 [file Table_1.docx]

Supplementary Material

# Supplementary Tables

Table 1 Differential expressed genes in Δ*abaI* strain

| GeneID | log2FoldChange(Δ*abaI*/WT) | Up-Down-Regulation(Δ*abaI*/WT) | Pvalue | Product |  |
| --- | --- | --- | --- | --- | --- |
| AUO97_RS06625 | -6.0744 | Down | 0 | acyl-CoA dehydrogenase | |
| AUO97_RS06615 | -5.9747 | Down | 0 | non-ribosomal peptide synthetase | |
| AUO97_RS06610 | -5.88657 | Down | 0 | outer membrane lipoprotein-sorting protein | |
| AUO97_RS06630 | -5.44259 | Down | 0 | acyl-CoA synthetase | |
| AUO97_RS06605 | -3.3445 | Down | 1.1E-107 | hypothetical protein | |
| AUO97_RS06600 | -2.88721 | Down | 4.97E-78 | alpha/beta hydrolase | |
| AUO97_RS06645 | -2.30926 | Down | 1.64E-07 | GNAT family N-acetyltransferase | |
| AUO97_RS06620 | -2.06687 | Down | 3.24E-06 | acyl carrier protein | |
| AUO97_RS11650 | -1.78566 | Down | 1.77E-32 | hemerythrin | |
| AUO97_RS06875 | -1.71559 | Down | 1.47E-59 | UDP-glucose 4-epimerase GalE | |
| AUO97_RS06595 | -1.64711 | Down | 1.23E-07 | phosphopantetheine-protein transferase | |
| AUO97_RS06865 | -1.58854 | Down | 5.17E-94 | L-lactate permease | |
| AUO97_RS06855 | -1.50567 | Down | 7.58E-53 | alpha-hydroxy-acid oxidizing enzyme | |
| AUO97_RS06860 | -1.45419 | Down | 3.24E-26 | transcriptional regulator LldR | |
| AUO97_RS11000 | -1.33315 | Down | 9.15E-17 | NADH-quinone oxidoreductase subunit K | |
| AUO97_RS06850 | -1.28401 | Down | 1.16E-24 | D-lactate dehydrogenase | |
| AUO97_RS11005 | -1.27855 | Down | 4.17E-96 | NADH-quinone oxidoreductase subunit L | |
| AUO97_RS10995 | -1.27133 | Down | 2.73E-39 | NADH-quinone oxidoreductase subunit J | |
| AUO97_RS11015 | -1.25693 | Down | 1.91E-85 | NADH-quinone oxidoreductase subunit N | |
| AUO97_RS10985 | -1.25538 | Down | 4.49E-80 | NADH-quinone oxidoreductase subunit H | |
| AUO97_RS11010 | -1.21787 | Down | 3.2E-73 | NADH-quinone oxidoreductase subunit M | |
| AUO97_RS10990 | -1.14605 | Down | 7.24E-42 | NADH-quinone oxidoreductase subunit I | |
| AUO97_RS04545 | -1.14342 | Down | 7.7E-49 | oxygen-dependent coproporphyrinogen oxidase | |
| AUO97_RS15040 | -1.13713 | Down | 4.45E-15 | cytosine permease | |
| AUO97_RS07490 | -1.11951 | Down | 1.42E-20 | AcrB/AcrD/AcrF family protein | |
| AUO97_RS12255 | -1.11535 | Down | 3.08E-46 | isocitrate lyase | |
| AUO97_RS04690 | -1.11028 | Down | 3.71E-16 | hypothetical protein | |
| AUO97_RS03915 | -1.09799 | Down | 1.5E-12 | GGDEF domain-containing protein | |
| AUO97_RS00400 | -1.09371 | Down | 8.57E-25 | U32 family peptidase | |
| AUO97_RS05665 | -1.08569 | Down | 0.000189 | MBL fold metallo-hydrolase | |
| AUO97_RS10980 | -1.06889 | Down | 2.64E-57 | NADH-quinone oxidoreductase subunit NuoG | |
| AUO97_RS07485 | -1.06411 | Down | 1.08E-09 | efflux RND transporter periplasmic adaptor subunit | |
| AUO97_RS05775 | -1.02016 | Down | 7.07E-23 | DUF2147 domain-containing protein | |
| AUO97_RS04585 | 1.001374 | Up | 1.2E-16 | putative DNA modification/repair radical SAM protein | |
| AUO97_RS11080 | 1.011794 | Up | 0.005496 | TetR/AcrR family transcriptional regulator | |
| AUO97_RS01645 | 1.013889 | Up | 1.31E-06 | heteromeric transposase endonuclease subunit TnsA | |
| AUO97_RS17070 | 1.014466 | Up | 0.011544 | muconolactone delta-isomerase | |
| AUO97_RS01845 | 1.025133 | Up | 1.34E-12 | Holliday junction branch migration protein RuvA | |
| AUO97_RS02620 | 1.033632 | Up | 8.86E-13 | outer membrane lipoprotein carrier protein LolA | |
| AUO97_RS13365 | 1.03403 | Up | 4.16E-23 | OmpA family protein | |
| AUO97_RS13625 | 1.034741 | Up | 7.53E-10 | hypothetical protein | |
| AUO97_RS11050 | 1.034749 | Up | 0.010909 | hypothetical protein | |
| AUO97_RS07280 | 1.037826 | Up | 0.003311 | GlsB/YeaQ/YmgE family stress response membrane protein | |
| AUO97_RS12195 | 1.042847 | Up | 2.75E-05 | SRPBCC domain-containing protein | |
| AUO97_RS05370 | 1.043606 | Up | 0.009118 | GNAT family N-acetyltransferase | |
| AUO97_RS16615 | 1.047928 | Up | 5.3E-50 | ImmA/IrrE family metallo-endopeptidase | |
| AUO97_RS01115 | 1.052979 | Up | 2.83E-17 | phosphate ABC transporter, permease protein PstA | |
| AUO97_RS14215 | 1.060287 | Up | 2.86E-13 | phenylacetate--CoA ligase | |
| AUO97_RS04650 | 1.061309 | Up | 4.72E-18 | SPOR domain-containing protein | |
| AUO97_RS10110 | 1.063226 | Up | 1.44E-20 | MacA family efflux pump subunit | |
| AUO97_RS03105 | 1.065265 | Up | 2.54E-05 | toxin |  |
| AUO97_RS16620 | 1.066128 | Up | 4.92E-29 | hypothetical protein | |
| AUO97_RS03210 | 1.07437 | Up | 1.36E-05 | VOC family protein | |
| AUO97_RS17840 | 1.076757 | Up | 2.31E-13 | sulfurtransferase TusD | |
| AUO97_RS12700 | 1.080796 | Up | 8.18E-05 | hypothetical protein | |
| AUO97_RS03445 | 1.084479 | Up | 0.001824 | DUF4184 domain-containing protein | |
| AUO97_RS12705 | 1.084642 | Up | 1.05E-10 | gamma-aminobutyraldehyde dehydrogenase | |
| AUO97_RS11550 | 1.084666 | Up | 1.11E-05 | metal-dependent hydrolase | |
| AUO97_RS01160 | 1.086385 | Up | 6.24E-17 | DUF541 domain-containing protein | |
| AUO97_RS15885 | 1.089957 | Up | 5.57E-27 | transcriptional regulator | |
| AUO97_RS06660 | 1.094631 | Up | 4.41E-06 | enoyl-CoA hydratase | |
| AUO97_RS19180 | 1.100387 | Up | 3.94E-07 | proline/glycine betaine transporter ProP | |
| AUO97_RS00970 | 1.100706 | Up | 0.002029 | PaaX family transcriptional regulator | |
| AUO97_RS13500 | 1.108039 | Up | 2.06E-05 | hypothetical protein | |
| AUO97_RS01405 | 1.109955 | Up | 5.76E-15 | membrane protein | |
| AUO97_RS17085 | 1.111993 | Up | 0.00072 | 3-oxoadipate CoA-transferase | |
| AUO97_RS13980 | 1.125222 | Up | 2.47E-07 | type VI secretion system baseplate subunit TssE | |
| AUO97_RS01255 | 1.130874 | Up | 1.28E-29 | LysR family transcriptional regulator | |
| AUO97_RS12355 | 1.132006 | Up | 3.99E-55 | META domain-containing protein | |
| AUO97_RS17615 | 1.135218 | Up | 1.36E-07 | universal stress protein | |
| AUO97_RS16430 | 1.135699 | Up | 9.01E-06 | CoA transferase subunit A | |
| AUO97_RS09015 | 1.136286 | Up | 1.71E-38 | FUSC family protein | |
| AUO97_RS14475 | 1.14236 | Up | 7.91E-09 | DNA polymerase V | |
| AUO97_RS02225 | 1.148054 | Up | 5.78E-38 | ABC transporter substrate-binding protein | |
| AUO97_RS01060 | 1.151745 | Up | 1.11E-10 | hypothetical protein | |
| AUO97_RS16600 | 1.153076 | Up | 4.64E-10 | hypothetical protein | |
| AUO97_RS14195 | 1.154194 | Up | 1.2E-05 | enoyl-CoA hydratase | |
| AUO97_RS00360 | 1.155982 | Up | 1.36E-07 | DNA-binding response regulator | |
| AUO97_RS06270 | 1.156561 | Up | 4.43E-10 | DUF2726 domain-containing protein | |
| AUO97_RS07670 | 1.165001 | Up | 4.24E-11 | aromatic amino acid transporter AroP | |
| AUO97_RS07665 | 1.166185 | Up | 1.94E-63 | fumarylacetoacetase | |
| AUO97_RS16010 | 1.168526 | Up | 1.13E-32 | haloacid dehalogenase | |
| AUO97_RS19190 | 1.176465 | Up | 1E-07 | SCPU domain-containing protein | |
| AUO97_RS19195 | 1.188775 | Up | 4.99E-16 | fimbrial biogenesis outer membrane usher protein | |
| AUO97_RS00020 | 1.189934 | Up | 0.005552 | hypothetical protein | |
| AUO97_RS16300 | 1.202481 | Up | 1.73E-45 | TIGR01244 family phosphatase | |
| AUO97_RS07165 | 1.204777 | Up | 0.005296 | hypothetical protein | |
| AUO97_RS10250 | 1.204815 | Up | 0.003704 | RidA family protein | |
| AUO97_RS02595 | 1.210701 | Up | 0.000229 | transcriptional regulator | |
| AUO97_RS07005 | 1.211821 | Up | 1.5E-37 | acyl-CoA desaturase | |
| AUO97_RS16305 | 1.212542 | Up | 1.27E-08 | sulfite exporter TauE/SafE family protein | |
| AUO97_RS12115 | 1.214564 | Up | 2.84E-32 | Fe-S biogenesis protein NfuA | |
| AUO97_RS01120 | 1.243207 | Up | 3.6E-18 | phosphate ABC transporter permease subunit PstC | |
| AUO97_RS14190 | 1.246926 | Up | 2.2E-06 | phenylacetate-CoA oxygenase/reductase subunit PaaK | |
| AUO97_RS13840 | 1.253491 | Up | 1.81E-20 | allophanate hydrolase | |
| AUO97_RS16595 | 1.264522 | Up | 4.69E-19 | PAAR domain-containing protein | |
| AUO97_RS19205 | 1.274509 | Up | 0.000646 | SCPU domain-containing protein | |
| AUO97_RS19200 | 1.27559 | Up | 4.91E-09 | molecular chaperone | |
| AUO97_RS00270 | 1.276369 | Up | 8.02E-05 | hypothetical protein | |
| AUO97_RS04580 | 1.307287 | Up | 1.03E-07 | hypothetical protein | |
| AUO97_RS15625 | 1.309804 | Up | 9.99E-06 | phage major capsid protein | |
| AUO97_RS12450 | 1.328101 | Up | 2.33E-19 | amino acid transporter | |
| AUO97_RS05515 | 1.333536 | Up | 1.76E-40 | NADPH:quinone oxidoreductase | |
| AUO97_RS03405 | 1.346309 | Up | 0.000218 | hypothetical protein | |
| AUO97_RS12825 | 1.375679 | Up | 2.99E-06 | crotonase | |
| AUO97_RS08885 | 1.376611 | Up | 0.000394 | DNA transfer protein p32 | |
| AUO97_RS00265 | 1.381641 | Up | 0.00074 | hypothetical protein | |
| AUO97_RS16295 | 1.396496 | Up | 6.32E-27 | MBL fold metallo-hydrolase | |
| AUO97_RS00890 | 1.398657 | Up | 7.72E-28 | hypothetical protein | |
| AUO97_RS04705 | 1.410219 | Up | 9.19E-16 | hypothetical protein | |
| AUO97_RS10870 | 1.454416 | Up | 1.2E-26 | flavin reductase | |
| AUO97_RS14180 | 1.4574 | Up | 3.51E-11 | phenylacetate-CoA oxygenase subunit PaaI | |
| AUO97_RS18630 | 1.457907 | Up | 3.68E-75 | aldehyde dehydrogenase | |
| AUO97_RS12715 | 1.458908 | Up | 1.83E-09 | aspartate aminotransferase family protein | |
| AUO97_RS17075 | 1.468197 | Up | 3.65E-14 | catechol 1,2-dioxygenase | |
| AUO97_RS12720 | 1.470222 | Up | 6.53E-08 | arginine N-succinyltransferase | |
| AUO97_RS01840 | 1.484748 | Up | 1.28E-65 | deoxyguanosinetriphosphate triphosphohydrolase | |
| AUO97_RS14170 | 1.487037 | Up | 1.93E-27 | 1,2-phenylacetyl-CoA epoxidase subunit A | |
| AUO97_RS05275 | 1.493798 | Up | 1.05E-18 | hypothetical protein | |
| AUO97_RS17080 | 1.496273 | Up | 1.16E-05 | 3-oxoadipate CoA-transferase subunit A | |
| AUO97_RS14175 | 1.534021 | Up | 1.12E-19 | 1,2-phenylacetyl-CoA epoxidase subunit B | |
| AUO97_RS01145 | 1.557012 | Up | 2.02E-49 | NAD-dependent aldehyde dehydrogenase | |
| AUO97_RS11225 | 1.567974 | Up | 2.97E-14 | trehalose-phosphatase | |
| AUO97_RS07745 | 1.607067 | Up | 3.22E-31 | DNA transfer protein p32 | |
| AUO97_RS17065 | 1.619212 | Up | 1.95E-10 | muconate cycloisomerase | |
| AUO97_RS14165 | 1.628035 | Up | 6.97E-17 | phenylacetic acid degradation bifunctional protein PaaZ | |
| AUO97_RS07095 | 1.660574 | Up | 6.92E-08 | C4-dicarboxylate ABC transporter | |
| AUO97_RS05520 | 1.685455 | Up | 2.39E-30 | TetR/AcrR family transcriptional regulator | |
| AUO97_RS18615 | 1.695967 | Up | 4.07E-46 | alcohol dehydrogenase | |
| AUO97_RS14185 | 1.739369 | Up | 1.42E-35 | phenylacetate-CoA oxygenase subunit PaaJ | |
| AUO97_RS14725 | 1.743263 | Up | 4E-17 | taurine ABC transporter substrate-binding protein | |
| AUO97_RS06395 | 1.744985 | Up | 1.2E-73 | MFS transporter | |
| AUO97_RS01125 | 1.794527 | Up | 4.22E-35 | phosphonate ABC transporter substrate-binding protein | |
| AUO97_RS00365 | 1.83163 | Up | 7.99E-22 | DNA-binding protein | |
| AUO97_RS13830 | 1.910832 | Up | 7.76E-13 | LamB/YcsF family protein | |
| AUO97_RS10865 | 1.926934 | Up | 5.8E-205 | methionine synthase | |
| AUO97_RS18900 | 1.984749 | Up | 6.85E-17 | hypothetical protein | |
| AUO97_RS14470 | 1.998418 | Up | 5.49E-06 | hypothetical protein | |
| AUO97_RS10860 | 2.07496 | Up | 2.1E-191 | DUF1852 domain-containing protein | |
| AUO97_RS07050 | 2.095124 | Up | 6.19E-12 | hypothetical protein | |
| AUO97_RS17150 | 2.097862 | Up | 5.78E-08 | nuclear transport factor 2 family protein | |
| AUO97_RS08715 | 2.116025 | Up | 7.01E-29 | EamA family transporter | |
| AUO97_RS19215 | 2.141387 | Up | 5.07E-60 | SCPU domain-containing protein | |
| AUO97_RS17165 | 2.147861 | Up | 7.66E-23 | amidase |  |
| AUO97_RS03425 | 2.174702 | Up | 4.1E-13 | hypothetical protein | |
| AUO97_RS00260 | 2.205577 | Up | 1.57E-67 | RtcB family protein | |
| AUO97_RS01130 | 2.216359 | Up | 6.67E-33 | aromatic amino acid transporter AroP | |
| AUO97_RS13835 | 2.236657 | Up | 1.63E-29 | DUF1445 domain-containing protein | |
| AUO97_RS17155 | 2.307109 | Up | 1.38E-08 | hypothetical protein | |
| AUO97_RS01135 | 2.335458 | Up | 2.75E-62 | pyruvate decarboxylase | |
| AUO97_RS08710 | 2.459453 | Up | 2.56E-34 | TetR/AcrR family transcriptional regulator | |
| AUO97_RS13825 | 2.587926 | Up | 3.04E-24 | divalent metal cation transporter | |
| AUO97_RS18910 | 2.644973 | Up | 3.5E-48 | hypothetical protein | |
| AUO97_RS17125 | 2.673537 | Up | 8.63E-24 | flavin reductase | |
| AUO97_RS17130 | 3.134825 | Up | 5.49E-24 | oxidoreductase | |
| AUO97_RS17135 | 3.137551 | Up | 8.58E-27 | KR domain-containing protein | |
| AUO97_RS17160 | 3.296468 | Up | 8.85E-48 | acyl-CoA dehydrogenase | |
| AUO97_RS17140 | 3.302273 | Up | 5.99E-17 | aromatic-ring-hydroxylating dioxygenase subunit beta | |
| AUO97_RS17145 | 3.557326 | Up | 8.79E-58 | aromatic ring-hydroxylating dioxygenase subunit alpha | |

Table 2 Differential expressed genes in Δ*abaR* strain

| GeneID | log2FoldChange(Δ*abaR*/WT) | Up-Down-Regulation(Δ*abaR*/WT) | Pvalue | Product |
| --- | --- | --- | --- | --- |
| AUO97_RS00540 | -1.46697 | Down | 7.01E-97 | elongation factor Ts |
| AUO97_RS00545 | -1.23446 | Down | 6.5E-101 | 30S ribosomal protein S2 |
| AUO97_RS01555 | -1.25779 | Down | 7.5E-12 | sulfate ABC transporter substrate-binding protein |
| AUO97_RS01560 | -1.60798 | Down | 9.23E-07 | alpha/beta hydrolase |
| AUO97_RS01565 | -1.80619 | Down | 3.15E-14 | sulfate ABC transporter permease subunit CysT |
| AUO97_RS01570 | -1.51136 | Down | 1.53E-08 | sulfate ABC transporter permease subunit CysW |
| AUO97_RS01575 | -1.55706 | Down | 1.89E-17 | sulfate ABC transporter ATP-binding protein |
| AUO97_RS01835 | -1.04633 | Down | 1.47E-50 | phosphoribosylformylglycinamidine synthase |
| AUO97_RS02870 | -1.24855 | Down | 7.89E-07 | septum formation inhibitor Maf |
| AUO97_RS03165 | -1.006 | Down | 1.04E-52 | translational GTPase TypA |
| AUO97_RS03220 | -1.02069 | Down | 2.64E-08 | DUF934 domain-containing protein |
| AUO97_RS03975 | -1.00367 | Down | 7.42E-47 | 50S ribosomal protein L13 |
| AUO97_RS04165 | -1.27941 | Down | 8.69E-08 | EamA/RhaT family transporter |
| AUO97_RS04275 | -1.36319 | Down | 2.43E-55 | 50S ribosomal protein L17 |
| AUO97_RS04280 | -1.26373 | Down | 9.25E-80 | DNA-directed RNA polymerase subunit alpha |
| AUO97_RS04285 | -1.14133 | Down | 7.65E-54 | 30S ribosomal protein S4 |
| AUO97_RS04290 | -1.09269 | Down | 1.17E-38 | 30S ribosomal protein S11 |
| AUO97_RS04305 | -1.29326 | Down | 1.32E-74 | preprotein translocase subunit SecY |
| AUO97_RS04310 | -1.09864 | Down | 1.39E-44 | 50S ribosomal protein L15 |
| AUO97_RS04320 | -1.15251 | Down | 1.55E-40 | 30S ribosomal protein S5 |
| AUO97_RS04325 | -1.14007 | Down | 2.33E-45 | 50S ribosomal protein L18 |
| AUO97_RS04330 | -1.03587 | Down | 5.13E-39 | 50S ribosomal protein L6 |
| AUO97_RS04335 | -1.12717 | Down | 1.21E-37 | 30S ribosomal protein S8 |
| AUO97_RS04340 | -1.00921 | Down | 2.04E-22 | 30S ribosomal protein S14 |
| AUO97_RS04345 | -1.04986 | Down | 2.5E-52 | 50S ribosomal protein L5 |
| AUO97_RS04360 | -1.33542 | Down | 2.89E-22 | 30S ribosomal protein S17 |
| AUO97_RS04365 | -1.38332 | Down | 9.06E-15 | 50S ribosomal protein L29 |
| AUO97_RS04370 | -1.30303 | Down | 6.38E-42 | 50S ribosomal protein L16 |
| AUO97_RS04375 | -1.38848 | Down | 3.29E-60 | 30S ribosomal protein S3 |
| AUO97_RS04380 | -1.32586 | Down | 3.99E-40 | 50S ribosomal protein L22 |
| AUO97_RS04385 | -1.2558 | Down | 3.56E-34 | 30S ribosomal protein S19 |
| AUO97_RS04390 | -1.25482 | Down | 9.97E-55 | 50S ribosomal protein L2 |
| AUO97_RS04395 | -1.33758 | Down | 3.92E-37 | 50S ribosomal protein L23 |
| AUO97_RS04400 | -1.14341 | Down | 4.11E-43 | 50S ribosomal protein L4 |
| AUO97_RS04405 | -1.19034 | Down | 3.39E-50 | 50S ribosomal protein L3 |
| AUO97_RS04410 | -1.18708 | Down | 2.7E-30 | 30S ribosomal protein S10 |
| AUO97_RS04430 | -1.0042 | Down | 1.6E-53 | flavohemoprotein |
| AUO97_RS04805 | -1.16847 | Down | 2.27E-41 | 50S ribosomal protein L19 |
| AUO97_RS05405 | -1.0155 | Down | 1.78E-11 | MFS transporter |
| AUO97_RS06420 | -1.08048 | Down | 2.86E-48 | ATP synthase epsilon chain |
| AUO97_RS06425 | -1.0439 | Down | 3.37E-50 | ATP synthase subunit beta |
| AUO97_RS06430 | -1.19299 | Down | 3.17E-60 | ATP synthase subunit gamma |
| AUO97_RS06435 | -1.00172 | Down | 5.3E-52 | ATP synthase subunit alpha |
| AUO97_RS06595 | -1.3648 | Down | 3.66E-06 | phosphopantetheine-protein transferase |
| AUO97_RS06600 | -2.11825 | Down | 8.13E-56 | alpha/beta hydrolase |
| AUO97_RS06605 | -2.58786 | Down | 4.19E-97 | hypothetical protein |
| AUO97_RS06610 | -4.02394 | Down | 0 | outer membrane lipoprotein-sorting protein |
| AUO97_RS06615 | -4.2825 | Down | 0 | non-ribosomal peptide synthetase |
| AUO97_RS06620 | -2.30484 | Down | 1.32E-05 | acyl carrier protein [Acinetobacter] |
| AUO97_RS06625 | -4.46229 | Down | 0 | acyl-CoA dehydrogenase |
| AUO97_RS06630 | -3.82819 | Down | 0 | acyl-CoA synthetase |
| AUO97_RS06635 | -3.91695 | Down | 1.44E-13 | LuxR family transcriptional regulator |
| AUO97_RS06645 | -1.18831 | Down | 0.010518 | GNAT family N-acetyltransferase |
| AUO97_RS06850 | -1.57625 | Down | 9.75E-39 | D-lactate dehydrogenase |
| AUO97_RS06855 | -1.66135 | Down | 2.76E-57 | alpha-hydroxy-acid oxidizing enzyme |
| AUO97_RS06860 | -1.89372 | Down | 8E-42 | transcriptional regulator LldR |
| AUO97_RS06865 | -1.80838 | Down | 1.65E-24 | L-lactate permease [Acinetobacter] |
| AUO97_RS07200 | -1.27639 | Down | 4.66E-18 | dual-action HEIGH metallo-peptidase |
| AUO97_RS08005 | -1.0278 | Down | 0.000671 | hypothetical protein |
| AUO97_RS08095 | -1.18969 | Down | 1.46E-36 | ATP-binding protein |
| AUO97_RS08100 | -1.00682 | Down | 4.07E-06 | hypothetical protein |
| AUO97_RS08105 | -1.50123 | Down | 4.68E-08 | type VI secretion protein |
| AUO97_RS08110 | -1.6293 | Down | 3.47E-44 | hypothetical protein |
| AUO97_RS08115 | -1.32724 | Down | 3.42E-11 | type VI secretion protein |
| AUO97_RS08120 | -1.78941 | Down | 6.98E-25 | hypothetical protein |
| AUO97_RS08125 | -1.63462 | Down | 1.71E-25 | conjugal transfer protein TrbI |
| AUO97_RS08130 | -1.53505 | Down | 1E-14 | hypothetical protein |
| AUO97_RS08135 | -1.78221 | Down | 2.31E-11 | conjugal transfer protein |
| AUO97_RS08175 | -1.63347 | Down | 2.54E-07 | hypothetical protein |
| AUO97_RS08180 | -1.43919 | Down | 5.89E-15 | hypothetical protein |
| AUO97_RS08185 | -1.2236 | Down | 5.97E-15 | type IV pili twitching motility protein PilT |
| AUO97_RS08195 | -1.35697 | Down | 2.13E-17 | hypothetical protein |
| AUO97_RS08200 | -1.00648 | Down | 0.000214 | hypothetical protein |
| AUO97_RS08205 | -1.03879 | Down | 1.12E-06 | hypothetical protein |
| AUO97_RS08345 | -1.0272 | Down | 1.39E-09 | hypothetical protein |
| AUO97_RS08400 | -3.96826 | Down | 3.8E-191 | IS5 family transposase |
| AUO97_RS08775 | -2.61617 | Down | 1.95E-12 | DNA-binding protein [Acinetobacter] |
| AUO97_RS08830 | -1.19333 | Down | 1.89E-65 | elongation factor Tu |
| AUO97_RS08850 | -1.12568 | Down | 8.29E-60 | 50S ribosomal protein L11 |
| AUO97_RS08855 | -1.15414 | Down | 4.74E-68 | 50S ribosomal protein L1 |
| AUO97_RS08860 | -1.25299 | Down | 6.63E-61 | 50S ribosomal protein L10 |
| AUO97_RS08865 | -1.29006 | Down | 8.87E-84 | 50S ribosomal protein L7/L12 [Acinetobacter] |
| AUO97_RS08870 | -1.03148 | Down | 2.36E-50 | DNA-directed RNA polymerase subunit beta |
| AUO97_RS08875 | -1.14418 | Down | 2.9E-73 | DNA-directed RNA polymerase subunit beta' |
| AUO97_RS09110 | -1.04917 | Down | 4.83E-48 | transcription termination/antitermination protein NusA |
| AUO97_RS09115 | -1.11845 | Down | 2.33E-57 | translation initiation factor IF-2 |
| AUO97_RS09120 | -1.02517 | Down | 3E-23 | ribosome-binding factor A |
| AUO97_RS09675 | -1.15939 | Down | 0.000133 | 50S ribosomal protein L28 [Moraxellaceae] |
| AUO97_RS10415 | -1.39769 | Down | 3.91E-09 | 50S ribosomal protein L35 |
| AUO97_RS10420 | -1.23068 | Down | 1.37E-68 | 50S ribosomal protein L20 [Acinetobacter] |
| AUO97_RS11000 | -1.10035 | Down | 4.37E-14 | NADH-quinone oxidoreductase subunit K [Acinetobacter] |
| AUO97_RS11015 | -1.00922 | Down | 9.05E-51 | NADH-quinone oxidoreductase subunit N |
| AUO97_RS11215 | -1.09784 | Down | 1.27E-35 | MFS transporter |
| AUO97_RS11345 | -1.00307 | Down | 1.11E-16 | 50S ribosomal protein L25 |
| AUO97_RS11530 | -1.12853 | Down | 2.42E-59 | 30S ribosomal protein S7 |
| AUO97_RS11535 | -1.22746 | Down | 1.66E-65 | elongation factor G |
| AUO97_RS11840 | -1.1171 | Down | 1.64E-58 | malate:quinone oxidoreductase |
| AUO97_RS12710 | -1.05094 | Down | 0.008596 | Lrp/AsnC family transcriptional regulator |
| AUO97_RS13535 | -1.23454 | Down | 0.000918 | transposase |
| AUO97_RS14515 | -1.77276 | Down | 0.000107 | amino acid ABC transporter permease [Acinetobacter] |
| AUO97_RS14525 | -1.13951 | Down | 1.92E-06 | ArtI protein [Acinetobacter] |
| AUO97_RS14730 | -1.41173 | Down | 1.07E-09 | taurine ABC transporter ATP-binding protein |
| AUO97_RS14735 | -1.63502 | Down | 9.12E-15 | taurine ABC transporter permease |
| AUO97_RS14740 | -2.01495 | Down | 7.36E-21 | taurine dioxygenase |
| AUO97_RS15190 | -1.4971 | Down | 0.000116 | hypothetical protein |
| AUO97_RS15195 | -4.0442 | Down | 0 | trifunctional transcriptional regulator/proline dehydrogenase/L-glutamate gamma-semialdehyde dehydrogenase |
| AUO97_RS15205 | -1.779 | Down | 1.1E-174 | sodium/proline symporter PutP |
| AUO97_RS16765 | -1.43715 | Down | 4.91E-05 | NAD(P)-dependent alcohol dehydrogenase |
| AUO97_RS17945 | -1.02093 | Down | 0.001295 | 3-dehydroquinate dehydratase |
| AUO97_RS18980 | -1.10603 | Down | 6.31E-29 | protoheme IX farnesyltransferase |
| AUO97_RS18985 | -1.2489 | Down | 4.3E-72 | 30S ribosomal protein S6 |
| AUO97_RS18990 | -1.16315 | Down | 5.79E-09 | 30S ribosomal protein S18 |
| AUO97_RS18995 | -1.41125 | Down | 8.4E-118 | 50S ribosomal protein L9 |
| AUO97_RS00020 | 1.222331 | Up | 0.016824 | hypothetical protein |
| AUO97_RS00035 | 1.135363 | Up | 0.000104 | glycosyl transferase |
| AUO97_RS00045 | 1.1511 | Up | 5.99E-08 | PIG-L family deacetylase |
| AUO97_RS00580 | 1.098532 | Up | 7.5E-35 | acyl-CoA dehydrogenase |
| AUO97_RS00665 | 1.639534 | Up | 6.49E-71 | alpha/beta hydrolase |
| AUO97_RS00945 | 1.245914 | Up | 5.65E-06 | hydrolase |
| AUO97_RS00985 | 1.091104 | Up | 0.000167 | DUF466 domain-containing protein |
| AUO97_RS00990 | 1.196287 | Up | 1.09E-52 | carbon starvation protein A |
| AUO97_RS01130 | 2.419967 | Up | 6.12E-42 | MFS transporter |
| AUO97_RS01135 | 2.254064 | Up | 1.16E-83 | ATP-dependent protease |
| AUO97_RS01145 | 1.668297 | Up | 1.74E-90 | NAD-dependent aldehyde dehydrogenase |
| AUO97_RS01330 | 1.013324 | Up | 2.48E-20 | hypothetical protein |
| AUO97_RS01525 | 1.258765 | Up | 1.74E-42 | PDZ domain-containing protein |
| AUO97_RS01635 | 1.134019 | Up | 0.017089 | NIF3 1 |
| AUO97_RS01855 | 1.056825 | Up | 5.66E-05 | amidohydrolase |
| AUO97_RS02080 | 1.096169 | Up | 2.03E-37 | amino acid permease |
| AUO97_RS02905 | 1.126299 | Up | 1.17E-16 | glutamine amidotransferase |
| AUO97_RS03090 | 1.25559 | Up | 7.25E-17 | DUF1508 domain-containing protein |
| AUO97_RS03105 | 1.492233 | Up | 1.04E-09 | toxin |
| AUO97_RS03160 | 1.158869 | Up | 6.14E-24 | large conductance mechanosensitive channel protein MscL |
| AUO97_RS03210 | 1.611018 | Up | 6.12E-14 | VOC family protein |
| AUO97_RS04000 | 1.156133 | Up | 1.9E-16 | acetyl/propionyl/methylcrotonyl-CoA carboxylase subunit alpha |
| AUO97_RS04005 | 1.052399 | Up | 4.02E-05 | enoyl-CoA hydratase |
| AUO97_RS04010 | 1.271182 | Up | 8.42E-15 | acyl-CoA dehydrogenase |
| AUO97_RS04015 | 1.648108 | Up | 9.91E-27 | acetyl-CoA carboxylase carboxyltransferase subunit |
| AUO97_RS04020 | 1.729121 | Up | 2.87E-19 | KR domain-containing protein |
| AUO97_RS04025 | 1.935767 | Up | 1.05E-53 | DUF1446 domain-containing protein |
| AUO97_RS04195 | 1.138211 | Up | 4.47E-65 | hypothetical protein |
| AUO97_RS04615 | 2.218472 | Up | 1.38E-19 | hypothetical protein |
| AUO97_RS04620 | 5.633865 | Up | 4.3E-106 | EamA/RhaT family transporter |
| AUO97_RS04905 | 1.264872 | Up | 2.7E-05 | hypothetical protein |
| AUO97_RS04915 | 1.231286 | Up | 4.4E-31 | LemA family protein |
| AUO97_RS05105 | 1.791924 | Up | 5.78E-67 | hypothetical protein |
| AUO97_RS05140 | 1.207876 | Up | 7.53E-08 | sulfate permease |
| AUO97_RS05215 | 1.821676 | Up | 9.5E-28 | EamA/RhaT family transporter |
| AUO97_RS05275 | 1.081392 | Up | 7.43E-10 | hypothetical protein |
| AUO97_RS05370 | 1.46563 | Up | 0.000574 | GNAT family N-acetyltransferase |
| AUO97_RS05440 | 1.02368 | Up | 2.04E-12 | hydrolase |
| AUO97_RS05450 | 1.550398 | Up | 9.3E-115 | NAD-dependent succinate-semialdehyde dehydrogenase |
| AUO97_RS05455 | 2.444237 | Up | 9.5E-158 | 4-aminobutyrate--2-oxoglutarate transaminase |
| AUO97_RS05465 | 2.866641 | Up | 1.4E-170 | amino acid permease |
| AUO97_RS05515 | 1.227311 | Up | 1.59E-21 | NADPH:quinone oxidoreductase |
| AUO97_RS05545 | 1.298715 | Up | 6.71E-05 | hypothetical protein |
| AUO97_RS05550 | 2.370634 | Up | 1.4E-202 | cation acetate symporter |
| AUO97_RS05555 | 1.922574 | Up | 6.03E-29 | DUF485 domain-containing protein [Acinetobacter] |
| AUO97_RS05595 | 2.656199 | Up | 3.7E-278 | acetate--CoA ligase |
| AUO97_RS05635 | 1.336995 | Up | 2.19E-84 | carbapenem susceptibility porin CarO |
| AUO97_RS05805 | 1.641335 | Up | 2.51E-71 | hypothetical protein |
| AUO97_RS06270 | 1.057684 | Up | 5.68E-08 | DUF2726 domain-containing protein |
| AUO97_RS06525 | 1.175945 | Up | 0.014654 | DUF3861 domain-containing protein |
| AUO97_RS06550 | 1.175091 | Up | 4.65E-05 | DoxX family protein |
| AUO97_RS06650 | 2.333258 | Up | 6.99E-28 | MFS transporter |
| AUO97_RS06655 | 2.75065 | Up | 7.52E-90 | enoyl-CoA hydratase/isomerase family protein |
| AUO97_RS06660 | 2.947701 | Up | 1.15E-61 | enoyl-CoA hydratase |
| AUO97_RS06665 | 2.788771 | Up | 2.4E-101 | acyl-CoA dehydrogenase |
| AUO97_RS06670 | 2.99023 | Up | 6.78E-31 | AMP-binding protein |
| AUO97_RS06675 | 2.626672 | Up | 1.8E-101 | 3-hydroxyisobutyrate dehydrogenase |
| AUO97_RS06680 | 2.412876 | Up | 5.5E-215 | methylmalonate-semialdehyde dehydrogenase (acylating) |
| AUO97_RS06695 | 1.738202 | Up | 2.05E-58 | amino acid permease |
| AUO97_RS06700 | 1.327951 | Up | 3.98E-13 | RidA family protein [Acinetobacter] |
| AUO97_RS06705 | 1.11741 | Up | 1.41E-34 | alanine racemase |
| AUO97_RS06710 | 1.417227 | Up | 3.65E-61 | D-amino-acid dehydrogenase |
| AUO97_RS06715 | 1.465739 | Up | 0.00052 | AsnC family transcriptional regulator [Acinetobacter] |
| AUO97_RS06785 | 1.075741 | Up | 0.002995 | sel1 repeat family protein |
| AUO97_RS06810 | 1.16987 | Up | 8.02E-09 | DUF4126 domain-containing protein |
| AUO97_RS06825 | 2.083996 | Up | 5.4E-190 | Fe/S-dependent 2-methylisocitrate dehydratase AcnD |
| AUO97_RS06830 | 2.154844 | Up | 7.5E-125 | 2-methylcitrate synthase |
| AUO97_RS06835 | 2.034936 | Up | 2.1E-108 | methylisocitrate lyase |
| AUO97_RS06840 | 1.166137 | Up | 1.01E-23 | GntR family transcriptional regulator |
| AUO97_RS07165 | 1.277517 | Up | 0.012728 | hypothetical protein |
| AUO97_RS07280 | 1.067484 | Up | 0.004097 | GlsB/YeaQ/YmgE family stress response membrane protein [Acinetobacter] |
| AUO97_RS07430 | 1.453655 | Up | 7.14E-70 | sodium-dependent transporter |
| AUO97_RS07590 | 1.141851 | Up | 1.13E-09 | PQQ-dependent sugar dehydrogenase |
| AUO97_RS07645 | 1.903493 | Up | 1.1E-213 | 4-hydroxyphenylpyruvate dioxygenase |
| AUO97_RS07655 | 1.885403 | Up | 5.1E-107 | VOC family protein |
| AUO97_RS07660 | 1.644119 | Up | 1.7E-102 | maleylacetoacetate isomerase |
| AUO97_RS07665 | 1.732833 | Up | 6.8E-139 | fumarylacetoacetase |
| AUO97_RS07670 | 2.082498 | Up | 2.6E-191 | aromatic amino acid transporter AroP |
| AUO97_RS07710 | 1.109972 | Up | 2.23E-40 | amino acid permease |
| AUO97_RS07715 | 1.277428 | Up | 7.06E-54 | imidazolonepropionase |
| AUO97_RS07720 | 1.392517 | Up | 2.47E-69 | formimidoylglutamase |
| AUO97_RS07725 | 1.165672 | Up | 1.52E-07 | DUF885 domain-containing protein |
| AUO97_RS07745 | 1.186045 | Up | 2.89E-16 | hypothetical protein |
| AUO97_RS08710 | 1.313813 | Up | 3.7E-08 | TetR/AcrR family transcriptional regulator |
| AUO97_RS08885 | 1.522507 | Up | 0.000292 | DNA transfer protein p32 |
| AUO97_RS08925 | 1.441162 | Up | 1.05E-45 | hypothetical protein |
| AUO97_RS09240 | 1.990121 | Up | 9.89E-17 | MerR family transcriptional regulator |
| AUO97_RS09745 | 1.159549 | Up | 2.63E-05 | hypothetical protein |
| AUO97_RS09845 | 1.430921 | Up | 1.56E-62 | phosphate acetyltransferase |
| AUO97_RS09850 | 1.495476 | Up | 5.58E-67 | acetate kinase |
| AUO97_RS10220 | 1.130523 | Up | 6.26E-63 | aconitate hydratase AcnA |
| AUO97_RS10285 | 1.368536 | Up | 9.08E-22 | hydroxypyruvate isomerase |
| AUO97_RS10375 | 1.815771 | Up | 7.89E-80 | acyl-CoA synthetase |
| AUO97_RS10780 | 1.238882 | Up | 5.12E-22 | acyl-CoA dehydrogenase |
| AUO97_RS10915 | 1.219079 | Up | 1.81E-60 | Ig-like domain |
| AUO97_RS11050 | 1.547215 | Up | 0.000298 | hypothetical protein |
| AUO97_RS11055 | 1.277695 | Up | 2.06E-85 | hypothetical protein |
| AUO97_RS11220 | 1.046402 | Up | 3.48E-27 | trehalose-6-phosphate synthase |
| AUO97_RS11270 | 1.287005 | Up | 3.58E-12 | threonylcarbamoyl-AMP synthase |
| AUO97_RS11550 | 1.451916 | Up | 2.38E-10 | metal-dependent hydrolase |
| AUO97_RS11750 | 1.160465 | Up | 3.76E-21 | gamma-glutamyltransferase family protein |
| AUO97_RS11975 | 1.267775 | Up | 0.006622 | cupin domain-containing protein [Acinetobacter] |
| AUO97_RS12450 | 1.001019 | Up | 9.88E-12 | amino acid transporter |
| AUO97_RS12700 | 1.891923 | Up | 1E-13 | hypothetical protein |
| AUO97_RS12705 | 1.395535 | Up | 2.52E-22 | gamma-aminobutyraldehyde dehydrogenase |
| AUO97_RS12715 | 2.700051 | Up | 1E-42 | aspartate aminotransferase family protein |
| AUO97_RS12720 | 2.456076 | Up | 4.22E-24 | arginine N-succinyltransferase |
| AUO97_RS12815 | 1.332388 | Up | 1.17E-05 | feruloyl-CoA synthase |
| AUO97_RS12825 | 1.944957 | Up | 1E-12 | crotonase |
| AUO97_RS12850 | 1.696962 | Up | 4.2E-09 | aromatic ring-hydroxylating dioxygenase subunit alpha |
| AUO97_RS12855 | 1.327513 | Up | 0.008247 | MFS transporter |
| AUO97_RS13000 | 1.063488 | Up | 0.004539 | hypothetical protein |
| AUO97_RS13005 | 1.167699 | Up | 0.003181 | hypothetical protein |
| AUO97_RS13050 | 1.254068 | Up | 0.011311 | hypothetical protein |
| AUO97_RS13365 | 1.816626 | Up | 1.1E-103 | OmpA family protein |
| AUO97_RS13440 | 1.28526 | Up | 2.37E-07 | aromatic acid/H+ symport family MFS transporter |
| AUO97_RS13450 | 1.198891 | Up | 0.000264 | KR domain-containing protein |
| AUO97_RS13465 | 1.354883 | Up | 1.45E-13 | benzoate 1,2-dioxygenase large subunit |
| AUO97_RS13485 | 1.043153 | Up | 3.76E-05 | Cu(I)-responsive transcriptional regulator [Acinetobacter] |
| AUO97_RS13760 | 1.143059 | Up | 9.1E-05 | MFS transporter |
| AUO97_RS13825 | 3.234393 | Up | 6.8E-158 | divalent metal cation transporter |
| AUO97_RS13830 | 3.201213 | Up | 2.6E-131 | LamB/YcsF family protein |
| AUO97_RS13835 | 3.473384 | Up | 8.5E-107 | DUF1445 domain-containing protein |
| AUO97_RS13840 | 2.743149 | Up | 1.4E-134 | allophanate hydrolase |
| AUO97_RS13845 | 2.961316 | Up | 3.2E-213 | ATP-grasp domain-containing protein |
| AUO97_RS14095 | 1.08262 | Up | 2.65E-11 | DUF333 domain-containing protein |
| AUO97_RS14165 | 1.53906 | Up | 3.3E-101 | phenylacetic acid degradation bifunctional protein PaaZ |
| AUO97_RS14170 | 1.032446 | Up | 2.82E-49 | 1,2-phenylacetyl-CoA epoxidase subunit A |
| AUO97_RS14175 | 1.3148 | Up | 1.55E-15 | 1,2-phenylacetyl-CoA epoxidase subunit B [Acinetobacter] |
| AUO97_RS14180 | 1.499868 | Up | 1.65E-67 | phenylacetate-CoA oxygenase subunit PaaI |
| AUO97_RS14185 | 1.536227 | Up | 1.89E-33 | phenylacetate-CoA oxygenase subunit PaaJ [Acinetobacter] |
| AUO97_RS14190 | 1.751782 | Up | 4.95E-74 | phenylacetate-CoA oxygenase/reductase subunit PaaK |
| AUO97_RS14195 | 1.762544 | Up | 1.76E-44 | enoyl-CoA hydratase |
| AUO97_RS14200 | 2.024242 | Up | 1.93E-52 | 2-(1,2-epoxy-1,2-dihydrophenyl)acetyl-CoA isomerase |
| AUO97_RS14205 | 1.999641 | Up | 3.01E-78 | 3-hydroxyacyl-CoA dehydrogenase |
| AUO97_RS14210 | 2.356772 | Up | 1.47E-86 | 3-oxoadipyl-CoA thiolase |
| AUO97_RS14215 | 2.48519 | Up | 1E-99 | phenylacetate--CoA ligase [Acinetobacter] |
| AUO97_RS14220 | 1.148118 | Up | 3.32E-18 | phenylacetic acid degradation operon negative regulatory protein PaaX |
| AUO97_RS14225 | 1.268769 | Up | 1.59E-07 | carbonic anhydrase |
| AUO97_RS14230 | 1.60666 | Up | 0.005131 | PaaI family thioesterase |
| AUO97_RS14350 | 2.142704 | Up | 3.8E-210 | hypothetical protein |
| AUO97_RS14360 | 2.522866 | Up | 4.7E-198 | hydroxymethylglutaryl-CoA lyase |
| AUO97_RS14365 | 2.454808 | Up | 4.6E-216 | acetyl/propionyl/methylcrotonyl-CoA carboxylase subunit alpha |
| AUO97_RS14370 | 2.29445 | Up | 1E-130 | enoyl-CoA hydratase |
| AUO97_RS14375 | 2.072343 | Up | 2.5E-231 | methylcrotonoyl-CoA carboxylase subunit beta |
| AUO97_RS14380 | 1.70927 | Up | 3.3E-142 | isovaleryl-CoA dehydrogenase [Acinetobacter] |
| AUO97_RS14385 | 1.473479 | Up | 6.7E-88 | TetR/AcrR family transcriptional regulator |
| AUO97_RS14390 | 1.584436 | Up | 2.3E-99 | AMP-binding protein |
| AUO97_RS14395 | 1.860351 | Up | 2.77E-29 | class I SAM-dependent methyltransferase |
| AUO97_RS14400 | 1.850229 | Up | 2.47E-24 | porin |
| AUO97_RS14470 | 1.887777 | Up | 0.000657 | hypothetical protein |
| AUO97_RS14480 | 1.066247 | Up | 1.68E-06 | LysE family translocator |
| AUO97_RS14690 | 1.049086 | Up | 3.4E-28 | cytochrome ubiquinol oxidase subunit I |
| AUO97_RS14855 | 1.419848 | Up | 5.24E-21 | peptide-methionine (R)-S-oxide reductase |
| AUO97_RS14970 | 1.54179 | Up | 9.37E-77 | amino acid ABC transporter substrate-binding protein |
| AUO97_RS14975 | 1.38723 | Up | 7.4E-36 | amino acid ABC transporter permease |
| AUO97_RS14980 | 1.428635 | Up | 7.26E-24 | amino acid ABC transporter permease |
| AUO97_RS14985 | 1.5406 | Up | 2.68E-41 | amino acid ABC transporter ATP-binding protein |
| AUO97_RS15145 | 1.837935 | Up | 2.31E-06 | hypothetical protein |
| AUO97_RS15625 | 1.100482 | Up | 0.000393 | phage major capsid protein |
| AUO97_RS16085 | 1.402496 | Up | 3E-06 | membrane protein |
| AUO97_RS16245 | 1.070249 | Up | 9.77E-14 | helix-turn-helix domain-containing protein |
| AUO97_RS16250 | 1.783306 | Up | 1.04E-38 | lipoyl synthase |
| AUO97_RS16255 | 2.613427 | Up | 5.1E-147 | thiamine pyrophosphate-dependent dehydrogenase E1 component subunit alpha [Acinetobacter] |
| AUO97_RS16260 | 2.751704 | Up | 4.6E-128 | alpha-ketoacid dehydrogenase subunit beta [Acinetobacter] |
| AUO97_RS16265 | 2.709018 | Up | 3.3E-146 | pyruvate dehydrogenase complex dihydrolipoamide acetyltransferase |
| AUO97_RS16270 | 2.482195 | Up | 1.3E-103 | dihydrolipoyl dehydrogenase |
| AUO97_RS16275 | 2.532405 | Up | 7.87E-35 | 3-oxoacyl-ACP reductase |
| AUO97_RS16280 | 2.044181 | Up | 8.99E-68 | 2,3-butanediol dehydrogenase [Acinetobacter] |
| AUO97_RS16305 | 1.320639 | Up | 5.63E-11 | sulfite exporter TauE/SafE family protein |
| AUO97_RS16380 | 1.063217 | Up | 1.95E-06 | MFS transporter |
| AUO97_RS16415 | 2.547582 | Up | 1.8E-236 | acetyl-CoA C-acyltransferase |
| AUO97_RS16420 | 3.111344 | Up | 1.4E-138 | hypothetical protein |
| AUO97_RS16425 | 3.545127 | Up | 5E-276 | CoA transferase subunit B |
| AUO97_RS16430 | 3.355226 | Up | 2.2E-212 | CoA transferase subunit A |
| AUO97_RS16465 | 1.065725 | Up | 1.2E-37 | GntP family permease |
| AUO97_RS16470 | 1.220277 | Up | 2.06E-27 | 3-hydroxybutyrate dehydrogenase |
| AUO97_RS16540 | 1.198637 | Up | 7.03E-05 | AdeA/AdeI family multidrug efflux RND transporter periplasmic adaptor subunit |
| AUO97_RS17065 | 1.072523 | Up | 0.000169 | muconate cycloisomerase |
| AUO97_RS17125 | 1.183404 | Up | 0.000615 | flavin reductase |
| AUO97_RS17130 | 1.630775 | Up | 5.91E-05 | oxidoreductase |
| AUO97_RS17135 | 1.379569 | Up | 0.000479 | KR domain-containing protein |
| AUO97_RS17140 | 2.112924 | Up | 7.35E-05 | aromatic-ring-hydroxylating dioxygenase subunit beta |
| AUO97_RS17145 | 1.870779 | Up | 2.13E-11 | aromatic ring-hydroxylating dioxygenase subunit alpha |
| AUO97_RS17155 | 1.445298 | Up | 0.005927 | hypothetical protein |
| AUO97_RS17160 | 1.417474 | Up | 1.19E-07 | acyl-CoA dehydrogenase |
| AUO97_RS17255 | 1.037498 | Up | 0.006082 | protocatechuate 3,4-dioxygenase subunit alpha |
| AUO97_RS17265 | 1.339034 | Up | 0.016976 | 4-carboxymuconolactone decarboxylase [Acinetobacter] |
| AUO97_RS17270 | 1.117003 | Up | 0.000133 | aromatic acid/H+ symport family MFS transporter |
| AUO97_RS17295 | 1.031416 | Up | 0.001354 | 3-oxoadipate CoA-transferase subunit A |
| AUO97_RS17510 | 1.418298 | Up | 7.41E-19 | hypothetical protein |
| AUO97_RS17615 | 1.348617 | Up | 1.88E-12 | univeal stress protein |
| AUO97_RS17805 | 1.595015 | Up | 5.1E-125 | D-amino acid dehydrogenase |
| AUO97_RS18020 | 1.379384 | Up | 0.016194 | hypothetical protein |
| AUO97_RS18065 | 1.120939 | Up | 0.01031 | hypothetical protein |
| AUO97_RS18165 | 1.293585 | Up | 0.013585 | hypothetical protein |
| AUO97_RS18270 | 1.483132 | Up | 0.007185 | hypothetical protein |
| AUO97_RS18455 | 1.352626 | Up | 3.22E-05 | RDD family protein |
| AUO97_RS18615 | 3.165241 | Up | 2.2E-227 | alcohol dehydrogenase |
| AUO97_RS18630 | 2.790133 | Up | 6.7E-289 | aldehyde dehydrogenase |
| AUO97_RS18635 | 1.045856 | Up | 1.34E-06 | ethanolamine permease |
| AUO97_RS18640 | 1.168491 | Up | 0.000154 | ethanolamine ammonia-lyase subunit EutB |
| AUO97_RS18860 | 2.626809 | Up | 4.9E-108 | AMP-binding protein |
| AUO97_RS18865 | 2.497141 | Up | 1.3E-129 | butyryl-CoA dehydrogenase |
| AUO97_RS18870 | 2.711438 | Up | 1.4E-155 | 3-hydroxyacyl-CoA dehydrogenase |
| AUO97_RS19040 | 1.091354 | Up | 0.000146 | hypothetical protein [Acinetobacter] |
| AUO97_RS19200 | 1.109533 | Up | 5.26E-07 | molecular chaperone |
| AUO97_RS19205 | 1.38997 | Up | 0.000595 | SCPU domain-containing protein |
| AUO97_RS19210 | 1.208871 | Up | 0.000104 | protein CsuA |
| AUO97_RS19215 | 2.042194 | Up | 3.42E-59 | SCPU domain-containing protein |

Table 3 Differential expressed genes in Δ*abaIR* strain

| GeneID | log2FoldChange(Δ*abaIR*/WT) | Up-Down-Regulation(Δ*abaIR*/WT) | Pvalue | Product |
| --- | --- | --- | --- | --- |
| AUO97_RS06625 | -4.810564081 | Down | 0 | acyl-CoA dehydrogenase |
| AUO97_RS06615 | -4.798341091 | Down | 0 | non-ribosomal peptide synthetase |
| AUO97_RS06610 | -4.684191859 | Down | 0 | outer membrane lipoprotein-sorting protein |
| AUO97_RS06630 | -4.479849984 | Down | 0 | acyl-CoA synthetase |
| AUO97_RS08905 | -3.443053657 | Down | 0 | membrane protein |
| AUO97_RS06635 | -3.169896529 | Down | 1.77E-13 | LuxR family transcriptional regulator |
| AUO97_RS06605 | -2.775977102 | Down | 1.2E-108 | hypothetical protein |
| AUO97_RS06600 | -2.592760202 | Down | 1.05E-77 | alpha/beta hydrolase |
| AUO97_RS06645 | -2.580535991 | Down | 5.26E-09 | GNAT family N-acetyltransferase |
| AUO97_RS06620 | -2.187695047 | Down | 8.04E-07 | acyl carrier protein |
| AUO97_RS10260 | -2.092687691 | Down | 1.5E-128 | NAD(P)(+) transhydrogenase (Re/Si-specific) subunit alpha |
| AUO97_RS10270 | -1.736715963 | Down | 1.03E-89 | NAD(P) transhydrogenase subunit beta |
| AUO97_RS10265 | -1.715456717 | Down | 5.71E-10 | NAD(P) transhydrogenase subunit alpha |
| AUO97_RS12875 | -1.610454816 | Down | 5.57E-10 | SDR family NAD(P)-dependent oxidoreductase |
| AUO97_RS06875 | -1.571786385 | Down | 1.63E-66 | UDP-glucose 4-epimerase GalE |
| AUO97_RS14380 | -1.571062479 | Down | 1.04E-66 | isovaleryl-CoA dehydrogenase |
| AUO97_RS13765 | -1.504167019 | Down | 1.89E-08 | DUF2147 domain-containing protein |
| AUO97_RS05565 | -1.467640693 | Down | 7.99E-23 | hypothetical protein |
| AUO97_RS12200 | -1.450434646 | Down | 1.71E-06 | esterase |
| AUO97_RS12630 | -1.447421801 | Down | 2.09E-77 | hypothetical protein |
| AUO97_RS05555 | -1.418971886 | Down | 2.68E-07 | DUF485 domain-containing protein |
| AUO97_RS14390 | -1.416019051 | Down | 6.55E-44 | AMP-binding protein |
| AUO97_RS14375 | -1.363301003 | Down | 1.35E-64 | methylcrotonoyl-CoA carboxylase subunit beta |
| AUO97_RS14395 | -1.333055716 | Down | 3.01E-07 | class I SAM-dependent methyltransferase |
| gene2510 | -1.254200121 | Down | 0.000113 | #N/A |
| AUO97_RS07705 | -1.221452269 | Down | 5.3E-91 | histidine ammonia-lyase |
| AUO97_RS06750 | -1.218989942 | Down | 6.02E-21 | short-chain dehydrogenase |
| AUO97_RS05160 | -1.216497945 | Down | 4E-69 | acetyl-CoA hydrolase |
| AUO97_RS12255 | -1.2055697 | Down | 7E-106 | isocitrate lyase |
| AUO97_RS05775 | -1.18255173 | Down | 1.22E-39 | DUF2147 domain-containing protein |
| AUO97_RS12620 | -1.159218991 | Down | 1.38E-62 | D-amino acid dehydrogenase |
| AUO97_RS10175 | -1.15568351 | Down | 2.52E-33 | hypothetical protein |
| AUO97_RS09645 | -1.148829981 | Down | 0.008712 | RNA-binding protein |
| AUO97_RS06840 | -1.146164239 | Down | 6.59E-12 | GntR family transcriptional regulator |
| AUO97_RS08910 | -1.136579054 | Down | 3.51E-05 | DUF1311 domain-containing protein |
| AUO97_RS10590 | -1.136034285 | Down | 3.57E-11 | ribosome-associated translation inhibitor RaiA |
| AUO97_RS12615 | -1.09534896 | Down | 1.37E-11 | RidA family protein |
| AUO97_RS06935 | -1.062020065 | Down | 2.24E-40 | DegT/DnrJ/EryC1/StrS family aminotransferase |
| AUO97_RS13875 | -1.053989883 | Down | 0.001534 | SRPBCC family protein |
| AUO97_RS14370 | -1.027361924 | Down | 1.64E-13 | enoyl-CoA hydratase |
| AUO97_RS15725 | -1.01932917 | Down | 6.13E-84 | malate synthase G |
| AUO97_RS09850 | -1.012081007 | Down | 3.26E-22 | acetate kinase |
| AUO97_RS07365 | -1.007776244 | Down | 2.76E-26 | RND transporter |
| AUO97_RS10915 | -1.00310968 | Down | 1.46E-42 | Ig-like domain |
| AUO97_RS14480 | 1.000516557 | Up | 3.19E-06 | LysE family translocator |
| AUO97_RS07010 | 1.0133961 | Up | 8.83E-37 | ferredoxin reductase |
| AUO97_RS01160 | 1.016484113 | Up | 3.04E-12 | DUF541 domain-containing protein |
| AUO97_RS14910 | 1.022615214 | Up | 0.010216 | hypothetical protein |
| AUO97_RS04145 | 1.027289495 | Up | 1.33E-12 | DUF3108 domain-containing protein |
| AUO97_RS13145 | 1.027636888 | Up | 0.000313 | DUF2184 domain-containing protein |
| AUO97_RS02595 | 1.050221533 | Up | 0.001537 | transcriptional regulator |
| AUO97_RS17175 | 1.059975124 | Up | 0.000226 | MFS transporter |
| AUO97_RS01125 | 1.065129674 | Up | 4.77E-12 | phosphonate ABC transporter substrate-binding protein |
| AUO97_RS05210 | 1.083855153 | Up | 7.7E-06 | XRE family transcriptional regulator |
| AUO97_RS04890 | 1.089851492 | Up | 5.25E-28 | bacterioferritin |
| AUO97_RS11550 | 1.102335386 | Up | 2.83E-06 | metal-dependent hydrolase |
| AUO97_RS00265 | 1.10618527 | Up | 0.00818 | hypothetical protein |
| AUO97_RS10860 | 1.109814353 | Up | 1.46E-41 | DUF1852 domain-containing protein |
| AUO97_RS13165 | 1.111887863 | Up | 0.008051 | hypothetical protein |
| AUO97_RS10765 | 1.117361686 | Up | 2.1E-12 | adenosine deaminase |
| AUO97_RS13135 | 1.12070687 | Up | 0.001552 | DUF2213 domain-containing protein |
| AUO97_RS12720 | 1.121710543 | Up | 5.81E-05 | arginine N-succinyltransferase |
| AUO97_RS04620 | 1.122555825 | Up | 0.002472 | EamA/RhaT family transporter |
| AUO97_RS07060 | 1.127777848 | Up | 0.001455 | sulfonate ABC transporter substrate-binding protein |
| AUO97_RS07165 | 1.134673245 | Up | 0.009058 | hypothetical protein |
| AUO97_RS10870 | 1.135437723 | Up | 5.34E-19 | flavin reductase |
| AUO97_RS12860 | 1.145646353 | Up | 0.002439 | outer membrane porin, OprD family |
| AUO97_RS05515 | 1.145856951 | Up | 1.85E-31 | NADPH:quinone oxidoreductase |
| AUO97_RS09015 | 1.161085465 | Up | 1.95E-46 | FUSC family protein |
| AUO97_RS06745 | 1.162966485 | Up | 0.001507 | DUF1003 domain-containing protein |
| AUO97_RS12715 | 1.16516071 | Up | 1.69E-06 | aspartate aminotransferase family protein |
| AUO97_RS05945 | 1.175348599 | Up | 6.55E-17 | replication protein C [Proteobacteria] |
| AUO97_RS11705 | 1.187305046 | Up | 8.43E-06 | hypothetical protein |
| AUO97_RS13005 | 1.19521274 | Up | 0.000815 | hypothetical protein |
| AUO97_RS07045 | 1.197457681 | Up | 5.63E-15 | hypothetical protein |
| AUO97_RS08880 | 1.200746597 | Up | 0.003007 | DNA transfer protein p32 |
| AUO97_RS14960 | 1.202220672 | Up | 0.000235 | SfnB family sulfur acquisition oxidoreductase |
| AUO97_RS18115 | 1.206540056 | Up | 0.00495 | stress-responsive nuclear envelope protein |
| AUO97_RS01255 | 1.211481854 | Up | 1.01E-39 | LysR family transcriptional regulator |
| AUO97_RS13835 | 1.218264558 | Up | 9.15E-09 | DUF1445 domain-containing protein |
| AUO97_RS07005 | 1.273658088 | Up | 3.63E-49 | acyl-CoA desaturase |
| AUO97_RS04575 | 1.274976219 | Up | 2.47E-25 | HPP family protein |
| AUO97_RS10250 | 1.279002189 | Up | 0.001758 | RidA family protein |
| AUO97_RS18125 | 1.290037253 | Up | 0.000228 | hypothetical protein |
| AUO97_RS12450 | 1.291462344 | Up | 6.13E-21 | amino acid transporter |
| AUO97_RS17065 | 1.294967434 | Up | 1.56E-06 | muconate cycloisomerase |
| AUO97_RS15625 | 1.311448775 | Up | 3.38E-06 | phage major capsid protein |
| AUO97_RS16845 | 1.31494929 | Up | 0.000124 | SMP-30/gluconolactonase/LRE family protein |
| AUO97_RS00195 | 1.319981098 | Up | 2.03E-07 | hypothetical protein |
| AUO97_RS01135 | 1.364315635 | Up | 2.3E-28 | pyruvate decarboxylase |
| AUO97_RS05215 | 1.376815835 | Up | 4.79E-15 | EamA/RhaT family transporter |
| AUO97_RS10110 | 1.390886913 | Up | 1.88E-28 | MacA family efflux pump subunit |
| AUO97_RS00260 | 1.406842394 | Up | 6.74E-23 | RtcB family protein |
| AUO97_RS08715 | 1.408770151 | Up | 1.15E-13 | EamA family transporter |
| AUO97_RS04705 | 1.424467059 | Up | 5.57E-05 | hypothetical protein |
| AUO97_RS05520 | 1.434908162 | Up | 6.18E-21 | TetR/AcrR family transcriptional regulator |
| AUO97_RS06395 | 1.455698678 | Up | 1.21E-48 | MFS transporter |
| AUO97_RS13175 | 1.463500264 | Up | 0.000157 | hypothetical protein |
| AUO97_RS17125 | 1.482712098 | Up | 2.03E-06 | flavin reductase |
| AUO97_RS18270 | 1.506892192 | Up | 0.0006 | hypothetical protein |
| AUO97_RS14730 | 1.525928785 | Up | 8.33E-26 | taurine ABC transporter ATP-binding protein |
| AUO97_RS08710 | 1.53094861 | Up | 9.1E-12 | TetR/AcrR family transcriptional regulator |
| AUO97_RS03405 | 1.583794725 | Up | 5.9E-06 | hypothetical protein |
| AUO97_RS17150 | 1.597978381 | Up | 8.73E-05 | nuclear transport factor 2 family protein |
| AUO97_RS13235 | 1.608502109 | Up | 0.000235 | hypothetical protein |
| AUO97_RS00970 | 1.610222783 | Up | 5E-07 | PaaX family transcriptional regulator |
| AUO97_RS19215 | 1.646501685 | Up | 1.18E-36 | SCPU domain-containing protein |
| AUO97_RS08885 | 1.666892923 | Up | 8.55E-06 | DNA transfer protein p32 |
| AUO97_RS16050 | 1.69031736 | Up | 8.39E-36 | sulfite exporter TauE/SafE family protein |
| AUO97_RS01130 | 1.762259496 | Up | 6.61E-20 | aromatic amino acid transporter AroP |
| AUO97_RS17165 | 1.797926772 | Up | 5.12E-16 | amidase |
| AUO97_RS17140 | 1.839119762 | Up | 2.78E-05 | aromatic-ring-hydroxylating dioxygenase subunit beta |
| AUO97_RS01560 | 1.905157138 | Up | 5.15E-26 | alpha/beta hydrolase |
| AUO97_RS17135 | 2.110174475 | Up | 2.41E-10 | KR domain-containing protein |
| AUO97_RS01555 | 2.113312655 | Up | 9.47E-77 | sulfate ABC transporter substrate-binding protein |
| AUO97_RS17130 | 2.203176565 | Up | 2.19E-10 | oxidoreductase |
| AUO97_RS18900 | 2.210965901 | Up | 6.94E-18 | hypothetical protein |
| AUO97_RS17160 | 2.239808823 | Up | 4.73E-22 | acyl-CoA dehydrogenase |
| AUO97_RS07050 | 2.241276362 | Up | 6.25E-14 | hypothetical protein |
| AUO97_RS17145 | 2.394634424 | Up | 3.66E-18 | aromatic ring-hydroxylating dioxygenase subunit alpha |
| AUO97_RS18910 | 2.455858156 | Up | 5.07E-14 | hypothetical protein |
| AUO97_RS03425 | 2.581083497 | Up | 1.35E-17 | hypothetical protein |
| AUO97_RS14725 | 2.601467607 | Up | 6.94E-65 | taurine ABC transporter substrate-binding protein |
